# Metabolomic Consequences of Genetic Inhibition of PCSK9 Compared With Statin Treatment

**DOI:** 10.1161/CIRCULATIONAHA.118.034942

**Published:** 2018-11-26

**Authors:** Eeva Sliz, Johannes Kettunen, Michael V. Holmes, Clare Oliver Williams, Charles Boachie, Qin Wang, Minna Männikkö, Sylvain Sebert, Robin Walters, Kuang Lin, Iona Y. Millwood, Robert Clarke, Liming Li, Naomi Rankin, Paul Welsh, Christian Delles, J. Wouter Jukema, Stella Trompet, Ian Ford, Markus Perola, Veikko Salomaa, Marjo-Riitta Järvelin, Zhengming Chen, Debbie A. Lawlor, Mika Ala-Korpela, John Danesh, George Davey Smith, Naveed Sattar, Adam Butterworth, Peter Würtz

**Affiliations:** 1 Center for Life Course Health Research (E.S., J.K., Q.W., S.S., M.-R.J., M.A.-K.), University of Oulu, Finland.; 2 Computational Medicine, Faculty of Medicine (E.S., J.K., Q.W., M.A.-K.), University of Oulu, Finland.; 3 Northern Finland Birth Cohorts, Faculty of Medicine (M.M.), University of Oulu, Finland.; 4 Biocenter Oulu, Oulu, Finland (E.S., J.K., Q.W., S.S., M.-R.J., M.A.-K.).; 5 Medical Research Council Population Health Research Unit (M.V.H.), University of Oxford, United Kingdom.; 6 Clinical Trial Service Unit and Epidemiological Studies Unit, Nuffield Department of Population Health (M.V.H., R.W., K.L., I.Y.M., R.C., Z.C.), University of Oxford, United Kingdom.; 7 National Institute for Health Research, Oxford Biomedical Research Centre, Oxford University Hospital, United Kingdom (M.V.H.).; 8 Medical Research Council Integrative Epidemiology Unit (M.V.H., D.A.L., M.A.-K., G.D.S.), University of Bristol, United Kingdom.; 9 Population Health Science, Bristol Medical School (D.A.L., M.A-.K., G.D.S.), University of Bristol, United Kingdom.; 10 MRC/BHF Cardiovascular Epidemiology Unit, Department of Public Health and Primary Care (C.O.W., J.D., A.B.), University of Cambridge, United Kingdom.; 11 Homerton College (C.O.W.), University of Cambridge, United Kingdom.; 12 National Institute for Health Research Blood and Transplant Research Unit in Donor Health and Genomics (J.D., A.B.), University of Cambridge, United Kingdom.; 13 Robertson Centre for Biostatistics (C.B., I.F.), University of Glasgow, United Kingdom.; 14 Institute of Cardiovascular and Medical Sciences (N.R., P.W., C.D., N.S.), University of Glasgow, United Kingdom.; 15 Systems Epidemiology, Baker Heart and Diabetes Institute, Melbourne, Victoria, Australia (Q.W., M.A.-K.).; 16 Department of Genomics of Complex Diseases, School of Public Health, Imperial College London, United Kingdom (S.S.).; 17 Chinese Academy of Medical Sciences, Beijing, China (L.L.).; 18 Department of Global Health, School of Public Health, Peking University, Beijing, China (L.L.).; 19 Department of Cardiology (J.W.J., S.T.), Leiden University Medical Center, The Netherlands.; 20 Department of Internal Medicine, Section of Gerontology and Geriatrics (S.T.), Leiden University Medical Center, The Netherlands.; 21 National Institute for Health and Welfare, Helsinki, Finland (M.P., V.S.).; 22 Institute for Molecular Medicine Finland, University of Helsinki (M.P.).; 23 University of Tartu, Estonian Genome Center, Estonia (M.P.).; 24 Department of Epidemiology and Biostatistics, MRC-PHE Centre for Environment and Health, Imperial College London, United Kingdom (M.R.-J.).; 25 Unit of Primary Care, Oulu University Hospital, Oulu, Finland (M.R.-J.).; 26 NMR Metabolomics Laboratory, School of Pharmacy, University of Eastern Finland, Kuopio (M.A.-K.).; 27 Department of Epidemiology and Preventive Medicine, School of Public Health and Preventive Medicine, Faculty of Medicine, Nursing and Health Sciences, The Alfred Hospital, Monash University, Melbourne, Victoria, Australia (M.A.-K.).; 28 Wellcome Trust Sanger Institute, Hinxton, United Kingdom (J.D.).; 29 Diabetes and Obesity Research Program, University of Helsinki, Finland (P.W.). Nightingale Health Ltd, Helsinki, Finland (P.W.).

**Keywords:** lipoproteins, Mendelian randomization analysis, metabolomics, statins

## Abstract

**Background:**

Both statins and proprotein convertase subtilisin/kexin type 9 (PCSK9) inhibitors lower blood low-density lipoprotein cholesterol levels to reduce risk of cardiovascular events. To assess potential differences between metabolic effects of these 2 lipid-lowering therapies, we performed detailed lipid and metabolite profiling of a large randomized statin trial and compared the results with the effects of genetic inhibition of PCSK9, acting as a naturally occurring trial.

**Methods:**

Two hundred twenty-eight circulating metabolic measures were quantified by nuclear magnetic resonance spectroscopy, including lipoprotein subclass concentrations and their lipid composition, fatty acids, and amino acids, for 5359 individuals (2659 on treatment) in the PROSPER (Prospective Study of Pravastatin in the Elderly at Risk) trial at 6 months postrandomization. The corresponding metabolic measures were analyzed in 8 population cohorts (N=72 185) using *PCSK9* rs11591147 as an unconfounded proxy to mimic the therapeutic effects of PCSK9 inhibitors.

**Results:**

Scaled to an equivalent lowering of low-density lipoprotein cholesterol, the effects of genetic inhibition of PCSK9 on 228 metabolic markers were generally consistent with those of statin therapy (*R*^2^=0.88). Alterations in lipoprotein lipid composition and fatty acid distribution were similar. However, discrepancies were observed for very-low-density lipoprotein lipid measures. For instance, genetic inhibition of PCSK9 had weaker effects on lowering of very-low-density lipoprotein cholesterol compared with statin therapy (54% versus 77% reduction, relative to the lowering effect on low-density lipoprotein cholesterol; *P*=2×10^-7^ for heterogeneity). Genetic inhibition of PCSK9 showed no significant effects on amino acids, ketones, or a marker of inflammation (GlycA), whereas statin treatment weakly lowered GlycA levels.

**Conclusions:**

Genetic inhibition of PCSK9 had similar metabolic effects to statin therapy on detailed lipid and metabolite profiles. However, PCSK9 inhibitors are predicted to have weaker effects on very-low-density lipoprotein lipids compared with statins for an equivalent lowering of low-density lipoprotein cholesterol, which potentially translate into smaller reductions in cardiovascular disease risk.

## Abstract 2

Supplemental Digital Content is available in the text.

**Clinical Perspective**

**What Is New?**

- Detailed lipoprotein lipid and metabolic effects of statin therapy in a large randomized, controlled trial are compared with the corresponding effects of proprotein convertase subtilisin/kexin type 9 (PCSK9) genetic inhibition in large population studies, acting as a naturally occurring trial of PCSK9 inhibitors.
- We demonstrate generally consistent effects of statins and PCSK9 genetic inhibition on a wide range of lipid-related metabolic markers when scaled to a similar lowering of low-density lipoprotein cholesterol.
- Differences are observed in lowering of very-low-density lipoprotein lipids and, more subtly, for the inflammation marker GlycA, with PCSK9 inhibition appearing to have a weaker effect in comparison with statins.

**What Are the Clinical Implications?**

- If very-low-density lipoprotein lipids have independent causal effects on cardiovascular disease risk, the observed discrepancy on very-low-density lipoprotein lipid lowering could contribute to differences in cardiovascular risk reductions between statins and PCSK9 inhibitors for an equivalent reduction in low-density lipoprotein cholesterol.
- The null associations on glycolysis-related measures and amino acids suggests that alternative mechanisms account for the association of genetic variants in *PCSK9* and risk of type 2 diabetes mellitus.
- These results exemplify the utility of large-scale metabolomic profiling with genetics and randomized trial data to uncover potential molecular differences between related therapeutics.

Statins are first line therapy to lower blood levels of low-density lipoprotein cholesterol (LDL-C) and reduce the risk of cardiovascular events.^1–3^ Treatment with proprotein convertase subtilisin/kexin type 9 (PCSK9) inhibitors has emerged as an additional effective therapy to lower LDL-C, resulting in reductions of ≈45% to 60%.^4,5^ Large cardiovascular outcome trials have recently demonstrated that PCSK9 inhibitors reduce the risk of major cardiovascular events when added to statin treatment.^6,7^ Based on the first major outcome trials,^6,7^ there has been some suggestions that PCSK9 inhibitors may be slightly less efficacious than statins for equivalent LDL-C reductions; however, other reports suggest that this is not the case, with apparent differences in cardiovascular event reduction explained by the short duration of the PCSK9 trials.^8^ Assessment of the detailed lipoprotein and other metabolic effects of statins and PCSK9 inhibitors could provide a more detailed understanding of these lipid-lowering therapies and shed light on potential differential effects on lipid metabolism.

The anticipated pharmacological effects of PCSK9 inhibitors may be assessed by LDL-C lowering alleles in the *PCSK9* gene, which act as unconfounded proxies for the lifetime effects of treatments.^9–11^ The observation of a prominent lower risk of coronary heart disease with LDL-C–lowering alleles in *PCSK9* was pivotal for accelerating the development of anti-PCSK9 therapeutics.^10^ Supporting the validity of using genetic proxies for molecular characterization of lipid-lowering targets, we have previously shown that LDL-C–lowering alleles in *HMGCR* (the gene encoding the target for statins) closely recapitulate the detailed metabolic changes associated with starting statin therapy in longitudinal cohorts, as assessed by nuclear magnetic resonance (NMR) metabolomics.^12^ These detailed metabolic effects of statins were recently confirmed in PREVEND IT (Prevention of Renal and Vascular End-stage Disease Intervention Trial), a small randomized trial.^13^ Other studies have assessed the associations of *PCSK9* variants with lipoprotein subclass profiles,^14,15^ and the treatment effects of PCSK9 inhibitors on lipoprotein particle concentrations and lipidomic measures have been examined in several small trials.^16–18^ However, prior studies have had limited power to assess potential differences between PCSK9 inhibition and statin therapy for equivalent reductions in LDL-C, complicating direct comparisons of their impact on detailed lipid and metabolite measures.

In the present study, we examined the effects of statin therapy and genetic inhibition of PCSK9 on a circulating profile of 228 metabolic measures, quantified by NMR metabolomics, including lipoprotein subclasses, their lipid concentrations and composition, fatty acid balance, and several nonlipid pathways. The metabolic effects of statin treatment were assessed in a large randomized, placebo-controlled trial. In the absence of NMR metabolomics data from a large randomized trial of PCSK9 inhibitor therapy, the anticipated pharmacological effects were examined for a loss-of-function variant in the *PCSK9* gene.^10,19^ Comparing the metabolomic effects of genetic inhibition of PCSK9 to statin therapy provides an opportunity to examine possible discrepancies in many circulating biomarkers, and in turn elucidate potential therapeutic differences in the molecular mechanisms to reduce cardiovascular risk.

## Methods

The authors declare that the summary statistics are available within the article and its online-only Data Supplement. The individual patient data analyzed in this study are available by application to the respective cohort committees.

### Study Design

**Figure 1.**
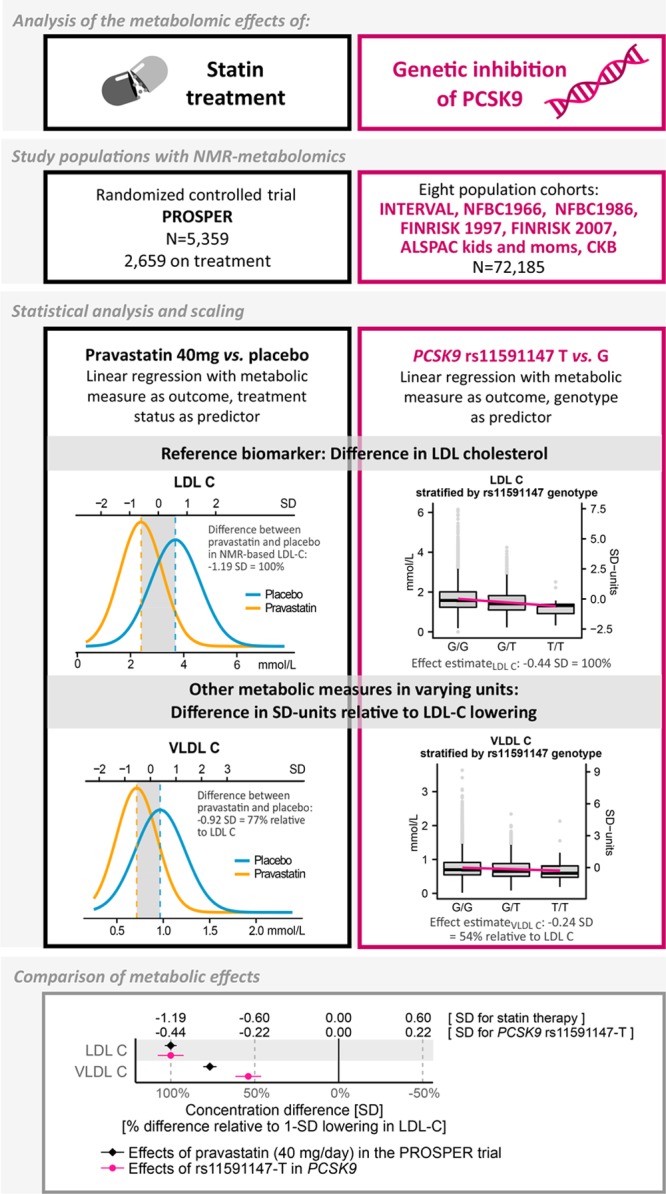
**Overview of the study design and statistical analyses.**

An overview of the study design is shown in Figure 1. NMR metabolomics was performed on 5359 blood samples from the PROSPER (Prospective Study of Pravastatin in the Elderly at Risk) trial^20^ at 6-month postrandomization, and 72 185 samples from eight population cohorts from the United Kingdom (INTERVAL,^21^ ALSPAC [Avon Longitudinal Study of Parents] mothers and offspring^22,23^), Finland (FINRISK-1997, FINRISK-2007, and Northern Finland Birth Cohort studies 1966 and 1986^24–26^), and China (China Kadoorie Biobank^27^). All study participants provided written informed consent, and study protocols were approved by the local ethics committees.

PROSPER is a double-blind, randomized, placebo-controlled trial investigating the benefit of pravastatin (40 mg/d) in elderly individuals at risk of cardiovascular disease, with 5804 participants (70–82 years old) from Scotland, Ireland, and The Netherlands enrolled between December 1997 and May 1999.^28^ All participants had above average plasma total cholesterol concentration (4.0–9.0 mmol/L) at baseline, and 50% had prior vascular disease. For the present study, 5359 samples (2659 on pravastatin) were measured by NMR metabolomics; all were previously unthawed 6-month postrandomization EDTA plasma samples stored at −80°C.^28^ Metabolite data from baseline samples were not available, however the randomization should ensure that there are limited between-group differences at baseline. Replication of the metabolic effects of pravastatin in PROSPER was done by comparison with recent results from PREVEND-IT.^13^

The metabolic effects of PCSK9 inhibition were assessed via the principle of Mendelian randomization using rs11591147-T (R46L), a loss-of-function allele robustly associated with lower LDL-C and decreased cardiovascular risk.^10,11^ The frequency of carriers of this effect allele was 2.2% (N=3135 carriers); clinical characteristics of these individuals are specified in Table I in the online-only Data Supplement. Additional genetic variants in the *PCSK9* locus, which have previously been used in Mendelian randomization studies on *PCSK9*,^11,29^ and display low linkage disequilibrium with rs11591147 (*R*^2^<0.2), were assessed in sensitivity analyses. To complement the comparison of *PCSK9* rs11591147-T effects against the statin trial, we further examined the metabolic effects of rs12916-T in *HMGCR* in the same study population, acting as a pseudotrial of a very small statin dose by naturally occurring randomization of HMG-CoA (HMG-coenzyme A) reductase inhibition.^12,30^ Among single nucleotide polymorphisms in *HMGCR*, rs12916 exhibits the strongest association with LDL-C and has been shown to affect hepatic *HMGCR* expression as well as cardiovascular risk.^11,12,30^ Last, to corroborate the validity of using genetic proxies to mimic the randomized trial effects, we compared metabolic effects of statin treatment in PROSPER with the corresponding effects of *HMGCR* rs12916-T. Pregnant women and individuals on lipid-lowering treatment were excluded from the analyses where information was available. Details of the cohorts are provided in Methods and Table I in the online-only Data Supplement.

### Lipid and Metabolite Quantification

High-throughput NMR metabolomics was used to quantify 228 lipoprotein lipids and polar metabolite measures from serum or plasma samples in the PROSPER trial and eight cohorts by the Nightingale platform (Nightingale Health Ltd, Helsinki, Finland). This provides simultaneous quantification of routine lipids, particle concentration, and lipid composition of 14 lipoprotein subclasses, abundant fatty acids, amino acids, ketones, and glycolysis-related metabolites in absolute concentration units (Table II in the online-only Data Supplement).^31^ The Nightingale NMR metabolomics platform has been widely used in epidemiological studies,^12,32,33^ and the measurement method has been previously described.^31–35^

### Statistical Analyses

The effects of statin therapy on the 228 metabolic measures in the PROSPER trial were assessed by comparing the mean metabolite concentrations in the treatment group with the placebo group at 6 months after randomization. The between-group difference in concentrations for each metabolic measure was quantified separately using linear regression with metabolite concentration as outcome and treatment status as predictor, adjusted for age and sex. All metabolite concentrations were scaled to standard deviation (SD) units before assessing the differences, to enable comparison of measures with different units and across wide ranges of concentration levels. Results in absolute units are presented in Table III in the online-only Data Supplement. The percentage differences in metabolite concentrations, relative to the placebo group, were examined as secondary analyses.

The effect of genetic inhibition of PCSK9 on each of the 228 metabolic measures was analyzed separately by fitting linear regression models with metabolite concentrations as outcome and rs11591147-T allele count as predictor, representing the number of LDL-C lowering alleles. For sensitivity analysis, we conducted equivalent tests of each metabolic measure with rs12916-T in *HMGCR* as the predictor. All genetic analyses assumed an additive effect and were adjusted for age, sex, and the first four genomic principal components. Effect sizes and standard errors from each cohort were combined using inverse variance-weighted fixed effect meta-analysis. All effect sizes were scaled to SD units of metabolite concentrations, as for analyses of PROSPER. The similarity between the overall patterns of metabolic effects attributable to PCSK9 inhibition and statin therapy was summarized using the linear fit of the effect estimates of 153 metabolic measures,^12^ covering all assayed measures except lipoprotein lipid ratios and 5 polar metabolites that could not be reliably quantified in PROSPER.

To facilitate comparison between the substantial metabolic effects of statin therapy with the smaller effects from genetic inhibition of PCSK9, results are presented relative to an equivalent (1-SD) lowering of LDL-C within each study design (as quantified by NMR metabolomics).^12,35^ For the statin trial, the estimates derived from comparing statin treatment with placebo were divided by 1.19 (because statins lowered LDL-C by 1.19 SD); for PCSK9 genetic associations, per-allele effect estimates were divided by 0.44; for sensitivity analyses using rs12916 in *HMGCR*, per-allele effect estimates were divided by 0.078. The scaling relative to LDL-C was used to interpret the reported effect sizes as a change in concentration in each metabolic measure (in SD units) that accompanies a 1-SD lowering of LDL-C by statin therapy and genetic inhibition of PCSK9. This scaling is in line with the principles of Mendelian randomization assuming that the genetic variants in *PCSK9* and *HMGCR* serve as instruments for the LDL-C exposure.

Although 228 metabolic measures in total were examined, the number of independent tests performed is lower because of the correlated nature of the measures.^35^ The number of independent tests was estimated by taking the number of principal components explaining 99% of the variation in the metabolic measures.^36^ Thus, significance was considered at *P*<0.0003 to account for the testing of 54 independent metabolic measures and 3 sets of analyses conducted (main effects of statins, *PCSK9*, and differences in their effects). The significance of differences in the effect estimates was determined using the formula below, and the corresponding *P* values were derived from the normal distribution:


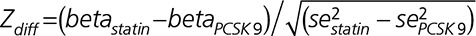


To facilitate visualization of the results, we focused on 148 measures that cover all the metabolic pathways assayed; results for the remaining measures are shown in Figure I in the online-only Data Supplement and Tables IV through VI in the online-only Data Supplement. Statistical analyses were conducted using R3.2 (www.r-project.org).

## Results

**Figure 2.**
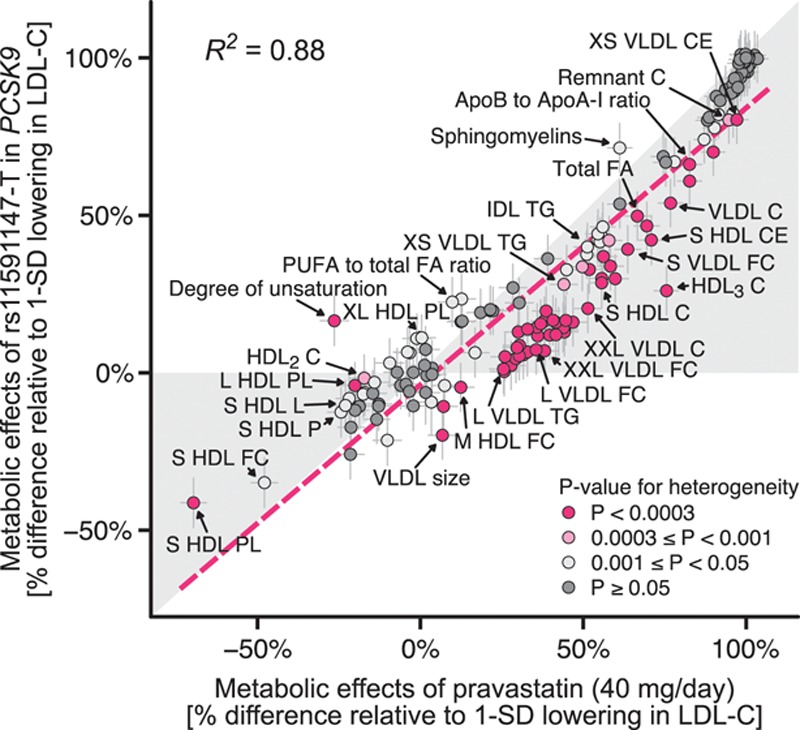
**Consistency of metabolic effects of statin treatment and *PCSK9* rs11591147-T.** The effect size of each metabolic measure is given with 95% confidence intervals in gray vertical and horizontal error bars. Color coding for the metabolic measure indicates the *P* value for heterogeneity between statin therapy and *PCSK9* rs11591147-T. *R*^2^ = 0.880 indicates goodness of fit (correlation squared). The red dashed line denotes the linear fit for the consistency of the metabolic effects (slope of this line = 0.879). C indicates cholesterol; FA, fatty acids; FC, free cholesterol; HDL, high-density lipoprotein; IDL, intermediate-density lipoprotein; LDL, low-density lipoprotein; PL, phospholipids; PUFA, polyunsaturated fatty acids; TG, triglycerides; and VLDL, very-low-density lipoprotein. A full list of metabolite names is given in Table II in the online-only Data Supplement.

Figure 1 provides an overview of the study design. Characteristics of the study populations are shown in the Table. Characteristics of each of the eight cohorts used for the genetic analyses (N=72 185) are provided separately in Table I in the online-only Data Supplement. The metabolic effects of statin treatment in PROSPER (pravastatin 40 mg/daily) and genetic inhibition of PCSK9 are compared in Figure 2. Overall, there was a high concordance of association of statin treatment and genetic inhibition of PCSK9 across the detailed metabolic profile (*R*^2^=0.88). Nonetheless, some discrepancies in effect sizes between statin treatment and *PCSK9* rs11591147 were evident, primarily for very-low-density lipoprotein (VLDL) lipids.

**Table.**
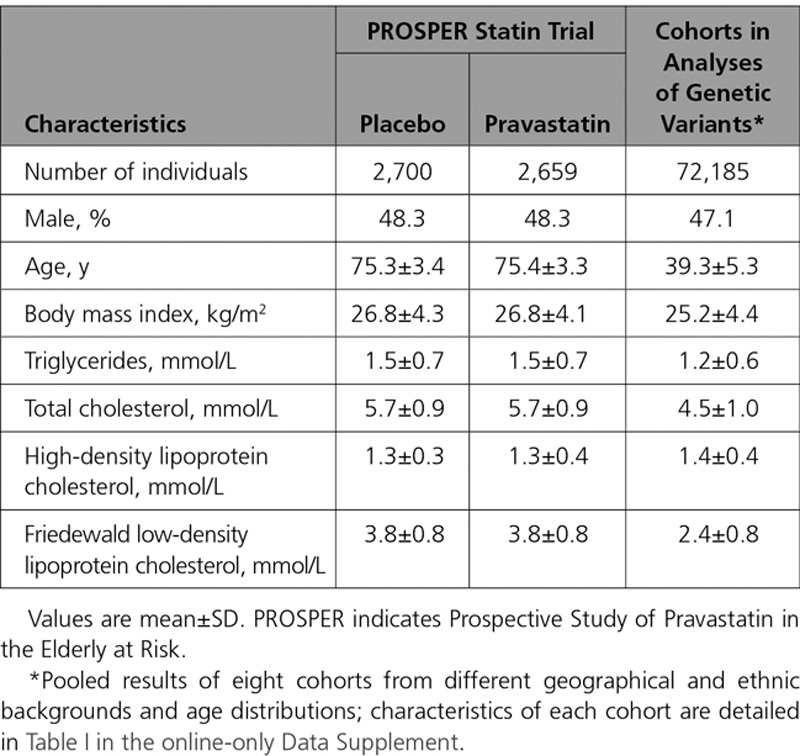
Baseline Characteristics of Participants in the PROSPER Statin Trial and Cohorts for Analyses of Genetic Inhibition of PCSK9

### Effects on Lipoprotein Lipids

**Figure 3.**
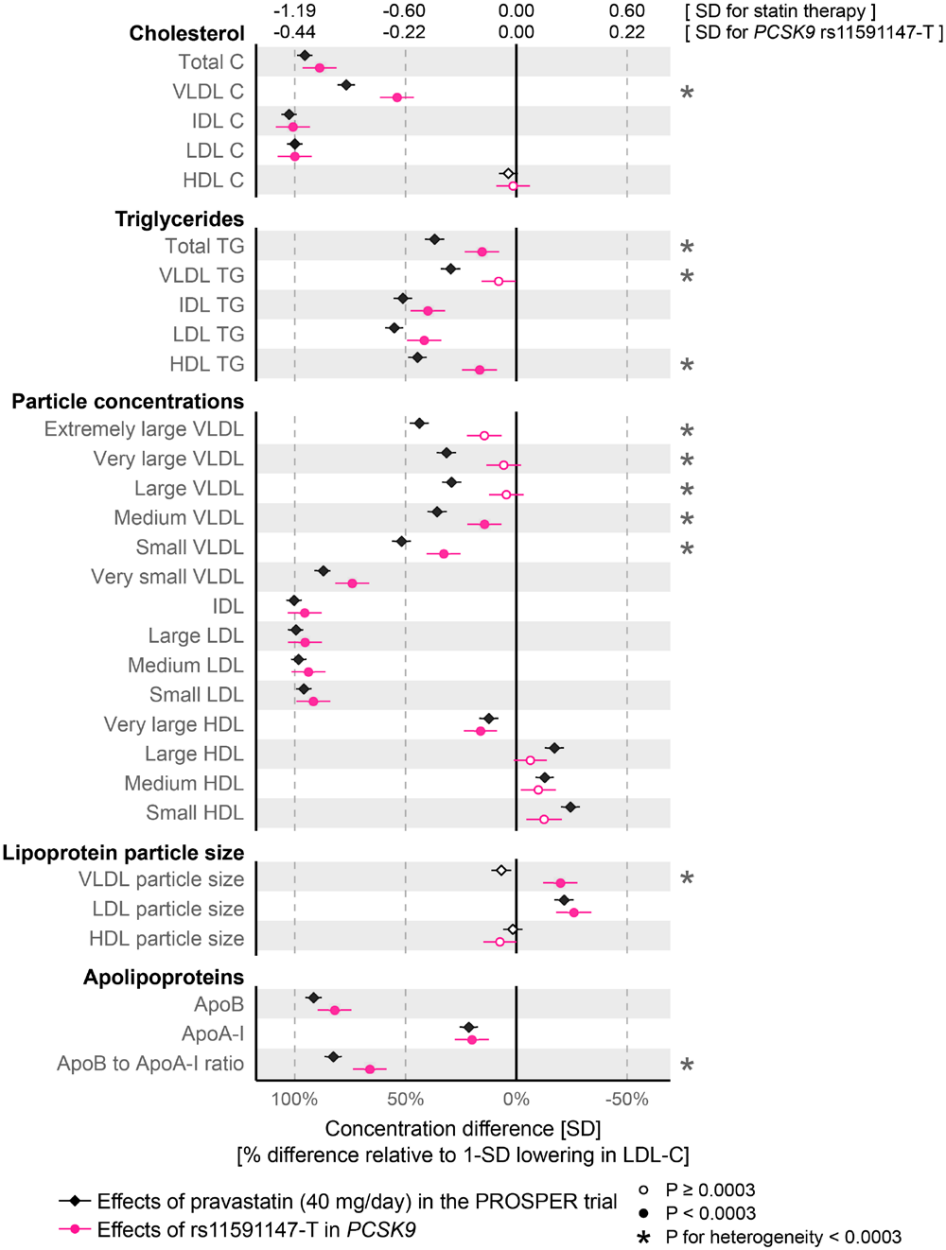
**Effects of statin treatment and genetic inhibition of PCSK9 on lipoprotein and lipid levels.** Differences in lipoprotein and lipid levels attributable to statin treatment were assessed in the PROSPER (Prospective Study of Pravastatin in the Elderly at Risk) trial at 6 months post randomization (black diamonds; n=5359 for which 2659 were on pravastatin 40 mg/d). The corresponding effects of *PCSK9* rs11591147-T were assessed for n=72 185 by meta-analysis of 8 cohorts (red circles). Error bars indicate 95% confidence intervals. Effect estimates are shown in SD-scaled concentration units (top axis) and relative to the lowering effect on low-density lipoprotein cholesterol (LDL-C; bottom axis). The results for different lipid types within the 14 lipoprotein subclasses are shown in Figure I in the online-only Data Supplement. Effects in absolute concentration units are listed in Table III in the online-only Data Supplement.

**Figure 4.**
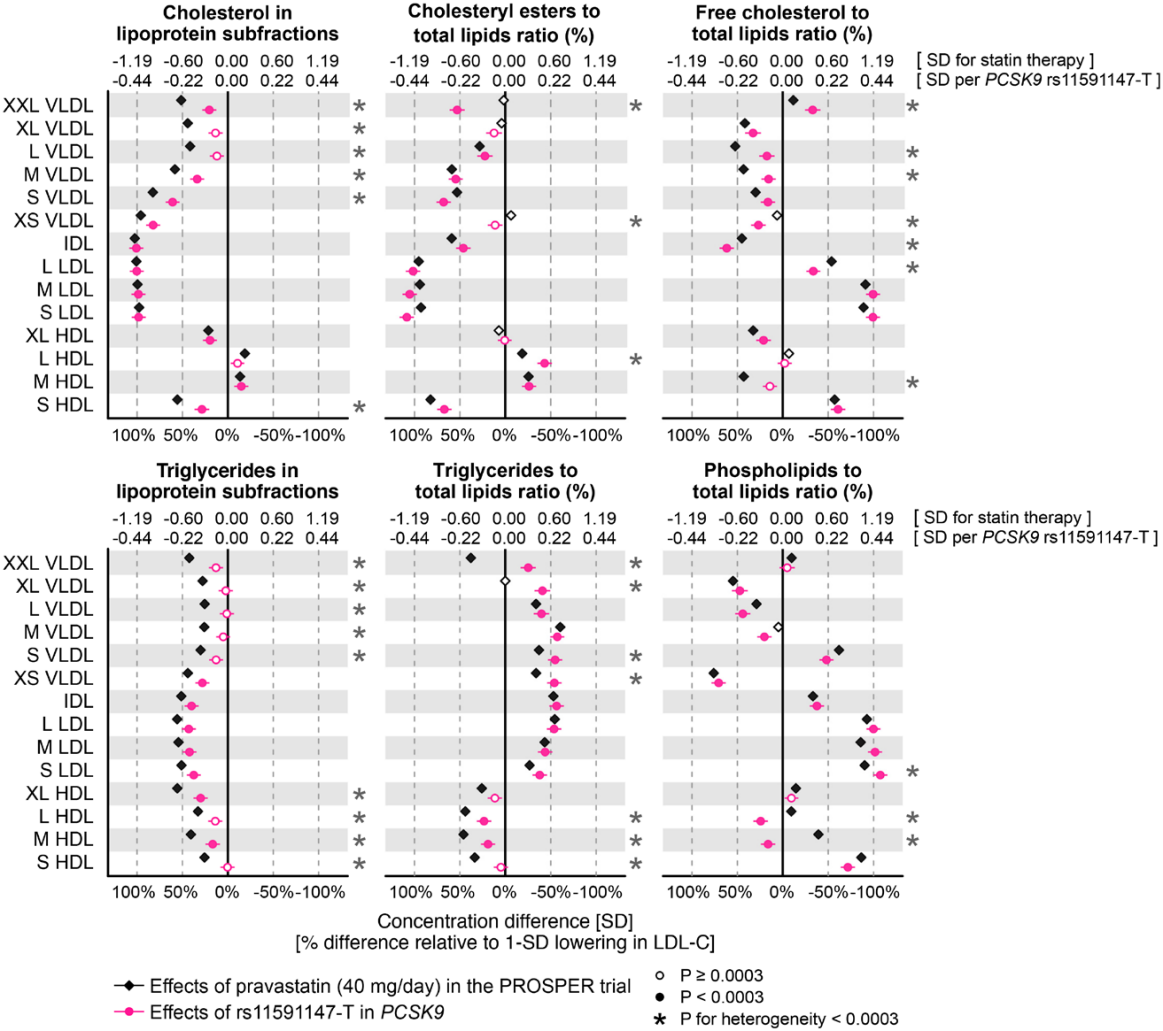
**Effects of statin treatment and genetic inhibition of PCSK9 on lipoprotein composition.** Differences in lipoprotein composition measures attributable to statin treatment were assessed 6 months post randomization in the PROSPER (Prospective Study of Pravastatin in the Elderly at Risk) trial (black). The corresponding effects of *PCSK9* rs11591147-T were assessed for n=72 185 (red). Error bars indicate 95% confidence intervals. Results are shown in SD-scaled concentration units (top axis) and relative to the lowering effect on low-density lipoprotein cholesterol (LDL-C; bottom axis).

The specific effects of statin therapy and genetic inhibition of PCSK9 on lipid fractions and 14 lipoprotein subclasses are shown in Figure 3. Scaled to the same lowering of LDL-C, *PCSK9* rs11591147 displayed similar effects as statin therapy for total cholesterol and intermediate-density lipoprotein cholesterol, with no effect on high-density lipoprotein cholesterol. However, *PCSK9* rs11591147 had a weaker effect on lowering VLDL cholesterol compared with statins (54% versus 77%, relative to the lowering effect on LDL-C [%_LDL-C_]; *P*_het_=2×10^-7^). These results were substantiated by the pattern of reduction in lipoprotein subclass particles: although the effects were similar for lowering particle concentrations in all 3 (small, medium, and large) LDL subclasses, the extent of lowering of small, medium-sized, and large VLDL particle concentrations was smaller for *PCSK9* rs11591147 compared with statin therapy. A similar discrepancy was observed for cholesterol concentrations within the 6 VLDL subclasses (Figure 4).

Both for statin therapy and genetic inhibition of PCSK9, the effects on triglyceride measures were modest compared with those observed for cholesterol levels in the same lipoprotein subfractions (Figure 3). The most pronounced lowering of triglycerides was seen for intermediate-density lipoprotein and LDL particles. For the equivalent reductions in LDL-C, *PCSK9* rs11591147 displayed a weaker effect than statin therapy on lowering total plasma triglycerides (16%_LDL-C_ versus 37%_LDL-C_; *P*_het_=3×10^-6^). Similar differences were seen for VLDL and high-density lipoprotein triglycerides. Consistent with the observed discrepancies for lowering of medium and large VLDL particles, genetic inhibition of PCSK9 resulted in modestly larger VLDL size, whereas statin therapy had no effect on this measure. The effects on apolipoprotein concentrations were broadly similar, albeit a larger decrease was observed with statins for the ratio of apolipoprotein B to A-I.

### Effects on Lipoprotein Composition

In addition to affecting the absolute lipid concentrations, both statin therapy and genetic inhibition of PCSK9 had prominent effects on the relative abundance of lipid types (free and esterified cholesterol, triglycerides, and phospholipids) in differently sized lipoprotein subclasses (Figure 4). The most pronounced lipoprotein composition effects were observed within LDL subclasses, with substantial lowering in the relative abundance of cholesteryl esters in LDL particles, alongside increases in the abundance of free cholesterol and phospholipids. These effects were very similar for statin treatment compared with *PCSK9* rs11591147 for the equivalent reductions in LDL-C. Subtle discrepancies between statin and genetic inhibition of PCSK9 were observed (eg, for the extent of lowering the fraction of free cholesterol in VLDL particles). The relative fraction of triglycerides in LDL and other apolipoprotein B–carrying particles increased similarly for both statins and *PCSK9* rs11591147, whereas statin therapy caused larger decreases in the relative abundance of triglycerides within high-density lipoproteins.

### Effects on Fatty Acids and Polar Metabolites

**Figure 5.**
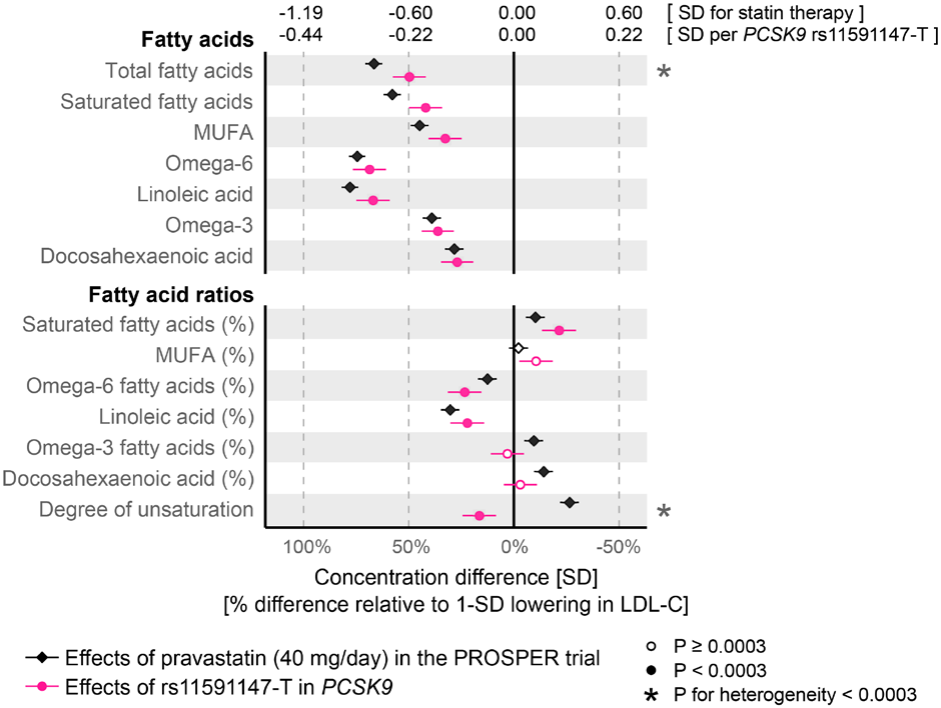
**Effects of statin treatment and genetic inhibition of PCSK9 on fatty acids.** Differences in fatty acid levels attributable to statin treatment were assessed 6 months post randomization in the PROSPER (Prospective Study of Pravastatin in the Elderly at Risk) trial (black). The corresponding effects of *PCSK9* rs11591147-T were assessed for n=72 185. Error bars indicate 95% confidence intervals. Results are shown in SD-scaled concentration units (top axis) and relative to the lowering effect on low-density lipoprotein cholesterol (LDL-C; bottom axis).

The effects of statin therapy and genetic inhibition of PCSK9 on fatty acid concentrations and the balance of fatty acid ratios are shown in Figure 5. Absolute concentrations of all fatty acids were lowered, with the most pronounced lowering for concentrations of linoleic acid, an omega-6 fatty acid commonly bound to cholesteryl esters in LDL particles. For the same lowering of LDL-C, the effects of statins and *PCSK9* rs11591147 were broadly similar, albeit with the lowering of total fatty acids being stronger in the case of statins (67%_LDL-C_ versus 50%_LDL-C_; *P*_het_=2×10^-4^). The effects on the fatty acid ratios were generally modest, both for statin therapy and *PCSK9* rs11591147. A pronounced discrepancy between these was observed for the overall degree of fatty acid unsaturation (16%_LDL-C_ reduction for *PCSK9* versus 26%_LDL-C_ increase for statin; *P*_het_=4×10^-20^).

**Figure 6.**
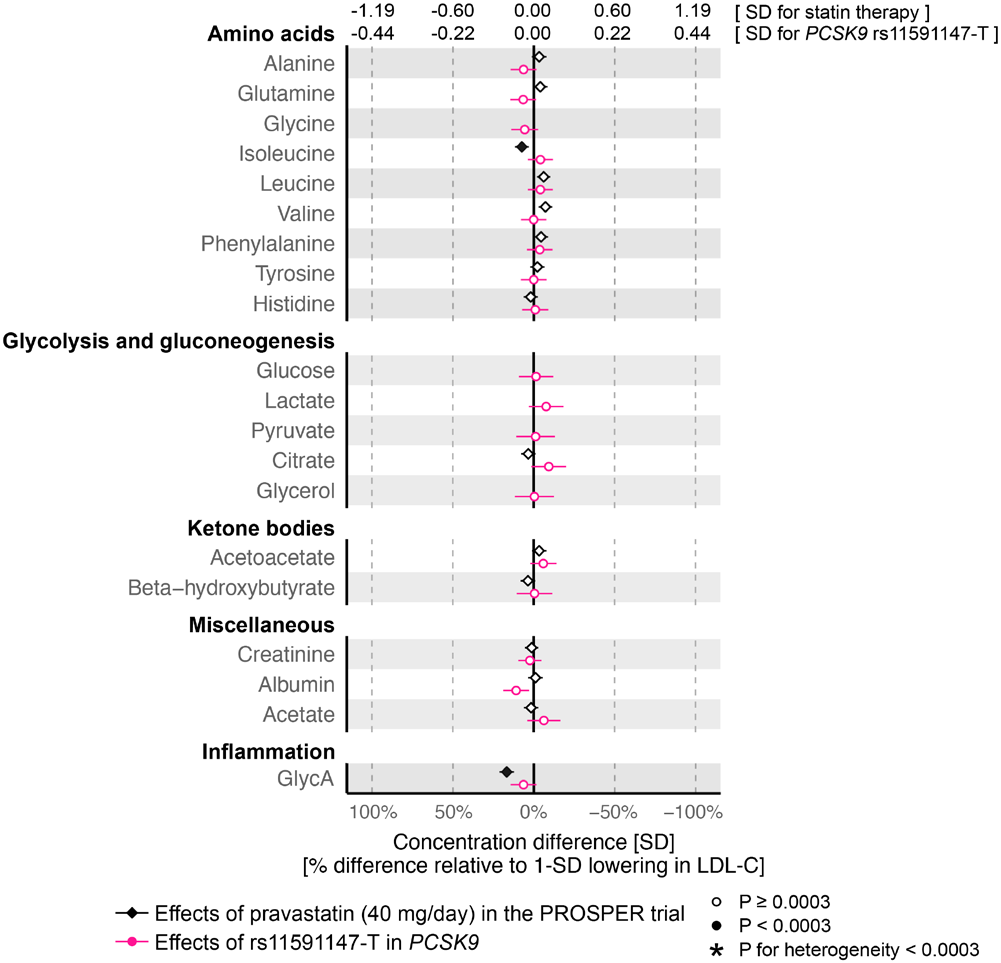
**Effects of statin treatment and genetic inhibition of PCSK9 on polar metabolites.** Differences in metabolite levels due to statin treatment were assessed 6 months post randomization in the PROSPER (Prospective Study of Pravastatin in the Elderly at Risk) trial (black). The corresponding effects of *PCSK9* rs11591147-T were assessed for n=72 185. Error bars indicate 95% confidence intervals. Glycine, glucose, lactate, pyruvate, and glycerol measures were not available from PROSPER. Results are shown in SD-scaled concentration units (top axis) and relative to the lowering effect on low-density lipoprotein cholesterol (LDL-C; bottom axis).

We further assessed the effects of statin therapy and *PCSK9* rs11591147 on polar metabolites and other metabolic measures quantified simultaneously in the metabolomics assay, including circulating amino acids, glycolysis metabolites, ketone bodies, and GlycA, a marker of chronic inflammation^37^ (Figure 6). Statin therapy caused only minor effects on these metabolic measures; the strongest lowering effects were observed for GlycA (17%_LDL-C_) and isoleucine (7%_LDL-C_). The effects of *PCSK9* rs11591147 were also very close to null for these measures, including for glycolysis related metabolites and markers of insulin resistance. Of note, information on glucose, lactate, and pyruvate were not available in the PROSPER trial because of glycolysis progression after sample collection.

### Comparison With PREVEND-IT Trial and Mendelian Randomization

To replicate the detailed metabolic effects of statins observed in PROSPER, we compared them with recent results from the PREVEND-IT trial obtained using the same NMR metabolomics platform.^13^ PREVEND-IT also examined the effects of pravastatin (40 mg/d) with metabolomic changes assessed from baseline to 3 months for 195 individuals on treatment. The detailed metabolic effects of statin treatment were highly concordant between PROSPER and PREVEND-IT (*R*^2^=0.96; Figure II in the online-only Data Supplement). When results were scaled to an equivalent lowering in LDL-C, 40 of 44 significant discrepancies observed between effects of *PCSK9* rs11591147 compared with PROSPER were similar or somewhat larger in PREVEND-IT, including the deviations in VLDL lipids; the only exceptions were 4 measures of lipoprotein composition (fraction of free cholesterol in XXL-VLDL, triglyceride fraction in XL-VLDL, triglyceride fraction in L-HDL, and phospholipid fraction in S-LDL; Figure I in the online-only Data Supplement).

We further compared the metabolic effects of *PCSK9* rs11591147 to those caused by rs12916 in the *HMGCR* gene, hereby using the genetic variants to effectively act as 2 naturally occurring trials in the same study population. The overall pattern of metabolic effects was highly similar for *PCSK9* rs11591147 and *HMGCR* rs12916 (*R*^2^=0.92; Figure IIIA in the online-only Data Supplement). Nonetheless, scaled to the equivalent LDL-C reductions, similar deviations were observed for VLDL lipids as when comparing *PCSK9* rs11591147 with the statin trial results (Figure IV in the online-only Data Supplement). Specifically, the lowering effect of *HMGCR* rs12916-T on particle concentrations of all VLDL subclasses was more similar to the effects of statin treatment than to those of *PCSK9* rs11591147-T, except in the case of very small VLDL. Differences were also observed for cholesterol and triglyceride concentrations in VLDL subclasses, whereas the lowering of total and saturated fatty acids was similar for the *HMGCR* and *PCSK9* variants. However, power to detect statistical differences on individual measures was modest, because of the much weaker LDL-C lowering effect of *HMGCR* rs12916. Last, the overall pattern of metabolic effects of statin therapy in PROSPER was highly concordant to effects of *HMGCR* rs12916 (*R*^2^=0.95; Figure IIIB in the online-only Data Supplement), signifying pharmacological and genetic inhibition of HMG-CoA reductase, respectively.

In sensitivity analyses, the pattern of metabolic effects from *PCSK9* rs11591147 was consistent across the cohorts (Figure V in the online-only Data Supplement). We also observed similar detailed patterns of metabolic effects as for rs11591147 when examining other genetic variants in *PCSK9* that have previously been used in Mendelian randomization studies^11,29^ (Figure VI in the online-only Data Supplement). Results for all the 228 metabolic measures quantified are illustrated in Figures I and IV in the online-only Data Supplement. Metabolic effects in absolute concentration units are listed in Table III in the online-only Data Supplement. The percentage differences in lipid and metabolite concentrations in the PROSPER statin trial are shown in Figure VII in the online-only Data Supplement. Effect estimates for all analyses are tabulated in Tables IV through VI in the online-only Data Supplement.

## Discussion

This study elucidates the comprehensive metabolic effects associated with statin therapy and PCSK9 inhibition. The results demonstrate that, in comparison with statin therapy, genetic inhibition of PCSK9 yields comparable changes across many different markers of lipid metabolism when scaled to the equivalent lowering of LDL-C. However, our results also suggest that PCSK9 inhibitors may be somewhat less efficacious at lowering VLDL particles. This could potentially contribute to subtle differences in potency for lowering cardiovascular disease for the equivalent reductions in LDL-C,^6^ because recent evidence suggests that VLDL cholesterol and other triglyceride-rich lipoprotein measures may causally contribute to the development of coronary heart disease independent of LDL-C.^38–40^ Moreover, trial data suggest that VLDL cholesterol is a stronger predictor of cardiovascular event risk than LDL-C among patients on statin therapy.^41,42^

Statins and PCSK9 inhibitors both lower circulating LDL-C levels via upregulation of LDL receptors on cell surfaces. Consistent with this shared mechanism for clearance of LDL particles, we found that statins and genetic inhibition of PCSK9 caused a highly consistent pattern of change across the detailed metabolic profile. The metabolomic profiling of the PROSPER trial corroborates previous studies with detailed measurements of metabolic effects of statin therapy, both as assessed in longitudinal cohorts and in a small randomized trial.^12,13^ By profiling a large number of individuals from multiple cohorts, our results also validate and extend previous studies examining the detailed metabolic effects of *PCSK9* rs11591147.^14,15^ Importantly, in relation to assessment of potential side-effects of PCSK9 inhibition, we did not observe effects on amino acids or other nonlipid metabolites, many of which are associated with risk of incident diabetes and cardiovascular events.^32,43,44^ Our results on the pattern of lowering VLDL particles attributable to genetic inhibition of PCSK9 are consistent with 2 small trials assessing the effects of the PCSK9 inhibitors alirocumab and evolocumab on lipoprotein particle concentrations; both trials showed substantial reductions in small and medium-sized VLDL particles, whereas the particle concentration of the large VLDL fraction was not affected.^16,17^ Similar results were also found in a small PCSK9-inhibitor trial using separation of VLDL subfractions and other lipid measures by ultracentrifugation, which also corroborate our results on a stronger effect on lowering of VLDL cholesterol in comparison with total plasma triglycerides.^18^ However, differences in assay methods complicate direct comparison of these trials to our results. Overall, these results provide orthogonal evidence for diverse lipoprotein lipid alterations by PCSK9 inhibitors, coherent with the comprehensive metabolic effects of statins.

Currently licensed PCSK9 inhibitors are given either instead of statins—when there is strong evidence of statin intolerance in those with familial hypercholesterolemia—or in addition to maximally tolerated statins in patients with existing vascular disease.^45^ Such treatment with PCSK9 inhibitors has been shown to be more efficacious in lowering LDL-C than the most potent statins.^4–6,8^ Mendelian randomization studies comparing *PCSK9* and *HMGCR* gene scores on cardiovascular outcomes have indicated nearly identical protective effects for equivalent reductions in LDL-C.^11,46^ However, when scaling the metabolic effects to an equivalent lowering in LDL-C, the results of the present study indicate subtle differences on multiple lipoprotein lipid measures. The most notable discrepancy was for VLDL lipids, suggesting weaker potency of PCSK9 inhibitors in clearance of these triglyceride-rich lipoproteins as compared with statins. These findings are supported by a recent study providing evidence that statins, but not PCSK9 inhibitors, improve triglyceride-rich lipoprotein metabolism after an oral fat load in normolipidemic men.^47^ The causal consequences of these differences in medium-sized and large VLDL particles, that are rich in triglycerides, remain unclear and warrant further investigation; whereas intermediate-density lipoprotein and the smallest VLDL particles can penetrate the arterial wall to cause atherosclerosis, it is commonly perceived not be to the case for larger VLDL particles.^38,48^ We also observed a difference in lowering of VLDL cholesterol levels; the cholesterol concentrations of VLDL particles are strongly associated with risk of myocardial infarction,^44^ and some studies have suggested that VLDL cholesterol could underpin the link between triglycerides and cardiovascular risk.^38,41^ If these VLDL particles do play a causal role in vascular disease, the discrepancy between statin therapy and PCSK9 inhibition could translate into slightly more potent cardiovascular risk reduction for the same LDL-C lowering for statins as compared with PCSK9 inhibition. We acknowledge that the present comparison of detailed metabolic effects of statin therapy and PCSK9 inhibition does not directly inform on the cardiovascular benefits of anti-PCSK9 therapies above current optimal care, but potentially in keeping with our findings, the cardiovascular outcome trials on PCSK9 inhibition have demonstrated slightly weaker cardiovascular event lowering compared with meta-analysis of statin trials per mmol/L reduction in LDL-C.^6^ Although potential explanations for this discrepancy include the short trial duration and choice of primary end point, other explanations, such as differences in anti-inflammatory effects, have also been suggested.^11,49^ Our results provide an additional hypothesis for exploration: the apparent weaker cardioprotective effects of PCSK9 inhibitors compared with statins per unit reduction in LDL-C may be attributable to weaker reductions in VLDL lipid concentrations by PCSK9 inhibition. This hypothesis warrants further investigation, including elucidation of the causal role of triglyceride-rich VLDL particles in tandem with further examinations of the detailed lipid effect of PCSK9 inhibitors.

Strengths and limitations of our study warrant consideration. The lack of NMR metabolomics data for a PCSK9 inhibition trial motivated the use of a loss-of-function variant in *PCSK9* as a proxy for the anticipated therapeutic effects. The close match in the detailed metabolic effects of statin therapy and *HMGCR* observed in this study substantiates the validity of using genetic variants to mimic lipid-lowering effects in randomized trial settings. Although we note that the metabolic profile of other statins may differ from that of pravastatin, the similarity between *HMGCR* and statin therapy that we identified provides reassurances about the generalizability of our findings to other statin types. To robustly assess the metabolic effects of genetic inhibition of PCSK9, we had >5 times the sample size of prior studies examining *PCSK9* rs11591147 on lipoprotein subclass profiles.^14,15^ Despite the large sample size, we had limited power to detect effects on glycolysis-related metabolites because of preanalytical effects causing depletion of glucose levels in the blood samples. Notwithstanding the increased risk for type 2 diabetes mellitus linked with *PCSK9* rs11591147-T, our results indicating minute effects on glycolysis traits are in line with larger studies reporting null effects on fasting glucose for this SNP. The divergency in VLDL lipid–lowering effects between statins and genetic inhibition of *PCSK9* could potentially be attributable to differences in the clinical characteristics of the older, high-risk patients of the PROSPER trial in comparison with the younger cohort participants included in the genetic analyses. However, similar VLDL lowering effects attributable to pravastatin as observed here in PROSPER were recently reported in PREVEND IT^13^ with younger and lower-risk trial participants (Figure I in the online-only Data Supplement). The differences in VLDL effects were also recapitulated when directly comparing the effects of genetic inhibition of PCSK9 to that of HMGCR in the same study population (Figure IV in the online-only Data Supplement), providing reassurance that the observed VLDL differences are primarily due to the molecular mechanisms. Furthermore, the scaling of results to the LDL-C lowering magnitude enables comparison of the metabolic effects regardless the possible differences in the absolute lipid levels between the study populations. A strength of the metabolomics platform used is the ability to profile lipoprotein subclasses and their lipid composition at high-throughput, however we acknowledge that other assays may provide even deeper characterization of lipid metabolism and nonlipid pathways to further clarify the molecular effects of lipid-lowering therapies.^50^

In conclusion, we found highly similar metabolic effects of statin therapy and genetic inhibition of PCSK9 across a comprehensive profile of lipids, lipoprotein subclasses, fatty acids, and polar metabolites. The detailed profiling of lipoprotein subclasses revealed weaker effects of PCSK9 inhibition on VLDL particles and their cholesterol concentrations in comparison with statins, when scaled to an equivalent lowering of LDL-C. If some of these VLDL lipids have independent causal effects on cardiovascular risk, this could contribute to subtle differences in cardiovascular event reduction between statins and PCSK9 inhibitors. More broadly, these results exemplify the utility of large-scale metabolomics in combination with randomized trials and genetics to uncover potential molecular differences between related therapeutics.

## Acknowledgments

The INTERVAL academic coordinating center receives core support from the UK Medical Research Council (G0800270), the British Heart Foundation (SP/09/002), the National Institute for Health Research, and Cambridge Biomedical Research Center, as well as grants from the European Research Council (268834), the European Commission Framework Program 7 (HEALTH-F2-2012–279233), Merck, and Pfizer. ALSPAC receives core support from the UK Medical Research Council and Wellcome Trust (Grant: 102215/2/13/2) and the University of Bristol. Northern Finland Birth Cohort studies received funding support (to S.S. and M.R.J.) by the European commission under Grant Agreement H2020-633595 for DynaHEALTH and H2020-733206 for LifeCycle; the Academy of Finland EGEA-project (GA-285547) and the Biocenter Oulu. The British Heart Foundation, UK Medical Research Council, and Cancer Research UK provide core funding to the Clinical Trial Service Unit and Epidemiological Studies Unit (CTSU) at the University of Oxford. DNA extraction and genotyping for the China Kadoorie Biobank was performed by BGI, Shenzhen, China. Drs Wang and Ala-Korpela work at The Baker Institute, which is supported in part by the Victorian Government’s Operational Infrastructure Support Program.

## Sources of Funding

This study was supported by the Academy of Finland (grant numbers 312476, 312477, 297338 and 307247), University of Oulu Graduate School, Strategic Research Funding from the University of Oulu, Finland, the Novo Nordisk Foundation (Grant Number NNF17OC0026062 and 15998), the Sigrid Juselius Foundation, and the UK Medical Research Council via the Medical Research Council University of Bristol Integrative Epidemiology Unit (MC_UU_12013/1 and MC_UU_12013/5). Dr Holmes is supported by a British Heart Foundation Intermediate Clinical Research Fellowship (FS/18/23/33512) and the National Institute for Health Research Oxford Biomedical Research Center. Dr Wang was supported by a Novo Nordisk Foundation Postdoctoral Fellowship (grant number NNF17OC0027034). Dr Rankin is supported by Glasgow Molecular Pathology NODE, which is funded by The Medical Research Council and The Engineering and Physical Sciences Research Council (MR/N005813/1). PROSPER metabolic profiling by NMR was supported by the European Federation of Pharmaceutical Industries Associations, Innovative Medicines Initiative Joint Undertaking, European Medical Information Framework grant number 115372, the European Commission under the Health Cooperation Work Program of the 7th Framework Program (Grant number 305507) “Heart ‘omics’ in AGEing” (HOMAGE). The INTERVAL study is funded by National Health Service Blood and Transplant (11-01-GEN) and has been supported by the National Institute for Health Research Blood & Transplant Research Units in Donor Health and Genomics (NIHR BTRU-2014–10024) at the University of Cambridge in partnership with National Health Service Blood and Transplant. NMR metablomics of the INTERVAL trial was funded by European Commission Framework Program 7 (HEALTH-F2-2012–279233). Data collection and metabolic profiling in the ALSPAC mother’s study were obtained from British Heart Foundation (SP/07/008/24066) and the Wellcome Trust (WT092830M). Genetic data in the ALSPAC mothers was obtained through funding from the Wellcome Trust (WT088806). ALSPAC offspring genetic data were obtained with support from 23andMe. The FINRISK studies have received financial support related to the present study from the National Institute for Health and Welfare, the Academy of Finland (139635), and the Finnish Foundation for Cardiovascular Research. Northern Finland Birth Cohort studies received funding support from University of Oulu Grant no. 65354, Oulu University Hospital Grant no. 2/97, 8/97, Ministry of Health and Social Affairs Grant no. 23/251/97, 160/97, 190/97, National Institute for Health and Welfare, Helsinki Grant no. 54121, Regional Institute of Occupational Health, Oulu, Finland Grant No. 50621, 54231. NFBC1986 received financial support from EU QLG1-CT-2000-01643 (EUROBLCS) Grant no. E51560, Nordic Academy for Advanced Study Grant no. 731, 20056, 30167, USA / NIHH 2000 G DF682 Grant no. 50945. The China Kadoorie Biobank baseline survey and first resurvey was supported by the Kadoorie Charitable Foundation in Hong Kong. Long-term follow-up has been supported by the UK Wellcome Trust (202922/Z/16/Z, 088158/Z/09/Z, 104085/Z/14/Z), National Key Research and Development Program of China (2016YFC0900500, 2016YFC0900501, 2016YFC0900504), Chinese Ministry of Science and Technology (2011BAI09B01), and National Natural Science Foundation of China (Grants No. 81390540, No. 81390541, No. 81390544). NMR metabolomics of China Kadoorie Biobank was supported by the British Heart Foundation Center of Research Excellence, Oxford (RE/13/1/30181).

## Disclosures

Dr Würtz is employee and shareholder of Nightingale Health Ltd, a company offering NMR-based metabolic profiling. Dr Kettunen reports stock options in Nightingale Health. The Clinical Trial Service Unit & Epidemiological Studies Unit (M.V.H., R.W., K.L., I.M., R.C., Z.C.) has received research grants from Abbott/ Solvay/Mylan, AstraZeneca, Bayer, GlaxoSmithKline, Merck, Novartis, Pfizer, Roche, and Schering. Dr Holmes has collaborated with Boehringer Ingelheim in research, and in accordance with the policy of the Clinical Trial Service Unit and Epidemiological Studies Unit (University of Oxford), did not accept any personal payment. Dr Salomaa has received a conference trip and an honorarium from Novo Nordisk. Dr Lawlor has received support from several government and charity health research funders and from Roche Diagnostics and Medtronic for research unrelated to that published here. Dr Sattar has consulted or been on the speaker bureau for AstraZeneca, Amgen, Sanofi, Boehringer Ingelheim, Janssen, Novo Nordisk and Eli-Lilly. He has also received funding from Boehringer Ingelheim. Dr Butterworth has received grants from Merck, Pfizer, Biogen, Bioverativ and AstraZeneca. The other authors report no conflicts.

## Supplementary Material
