# Supplementary material for: Metabolomic Consequences of Genetic Inhibition of PCSK9 Compared With Statin Treatment
Source: Circulation. 2018 Nov 26;138(22):2499–512. doi: 10.1161/CIRCULATIONAHA.118.034942 (PMC6254781; doi:10.1161/CIRCULATIONAHA.118.034942)
Supplement: Supplementary file 1 [file cir-138-2499-s001.pdf]

SUPPLEMENTAL MATERIAL

Metabolomic consequences of genetic inhibition of PCSK9 compared with statin treatment

Sliz, Kettunen, Holmes *et al*.

Contents

Supplementary Methods 1

Table S1. Clinical characteristics of the eight cohorts included in the genetic analyses of *PCSK9* rs11591147 and *HMGCR* rs12916 7

Table S2. Metabolite mean (SD) concentrations in PROSPER and cohorts used for genetic analyses 9

Figure S1. Effects of statin treatment and genetic inhibition of PCSK9 on 228 metabolic traits. 17

Figure S2. Consistency of metabolic effects of pravastatin 40 mg/day in PROSPER vs. PREVEND IT trials. ... 17

Figure S3. Consistency of metabolic effects of *HMGCR* rs12916-T vs. (A) *PCSK9* rs11591147-T, and (B) statin therapy in the PROSPER trial. 19

Figure S4. Metabolomic effects of statin treatment, *HMGCR* rs12916-T and *PCSK9* rs11591147-T. 20

Figure S5. Consistency of metabolomic effects of *PCSK9* rs11591147-T across the cohorts on 228 metabolic traits.. 21

Figure S6. Consistency of metabolic effects of the main instrument *PCSK9* rs11591147-T with other *PCSK9* variants. 23

Figure S7. Percentage differences in metabolite concentrations in pravastatin versus placebo group in PROSPER trial. 24

Summary statistics are provided in a separate Excel spreadsheet file:

Table S3. Main results in absolute concentration units.

Table S4. Metabolic effects of statin therapy in the PROSPER trial in SD-units.

Table S5. Metabolic effects of *PCSK9* rs11591147-T in SD units.

Table S6. Metabolic effects of *HMGCR* rs12916-T in SD-units.

Supplementary Methods

RANDOMIZED STATIN TRIAL

The PROspective Study of Pravastatin in the Elderly at Risk (PROSPER) trial design has been published previously.[1] In brief, 5,804 elderly adults (70–82 years old) were enrolled. This was a double-blind, randomized placebo-controlled trial investigating the benefit of pravastatin (40 mg/day) in elderly individuals at risk of cardiovascular disease. Participants were identified in the primary care setting from three centres: Glasgow, Scotland; Cork, Ireland and Leiden, the Netherlands. All participants had high-normal to high cholesterol concentration (4.0 to 9.0 mmol/L) at baseline. Additionally, 50% of patients had evidence of vascular disease (physician diagnosed stable angina, stroke, transient ischemic attack or myocardial infarction) and the remaining 50% of patients had high risk of vascular disease as they had either hypertension, diabetes or were smokers. Individuals with congestive heart failure were excluded. The primary outcome measure of PROSPER was a composite cardiovascular outcome. Patients were recruited between December 1997 and May 1999. The investigation conforms with the principles outlined in the Declaration of Helsinki.

Fasting venous blood samples were collected at baseline and at 3-month intervals and biobanked at -80°C. For the present study, previously unthawed 6-month post-randomisation samples were used, with metabolic profile quantified by NMR metabolomics (Nightingale Health Ltd Helsinki, Finland; 2014 quantification version) for 5,359 samples (2659 on pravastatin).[2] Some metabolites are missing due to EDTA plasma (glycine, glycerol, pyruvate) or post-sample draw glycolysis progression (glucose, lactate) flagged by the automated quality control analyses of the metabolite quantification.

The institutional ethics review boards of all three European centres approved the study protocol.[3] All participants provided written informed consent to participate in the study and for long-term follow-up.

POPULATION COHORTS USED FOR GENETIC ANALYSES OF PCSK9 AND HMGCR

Supplementary Table 1 describes the characteristics of the cohort studies included in the genetic analysis. The participating studies are detailed below. The total number of participants included in the genetic analyses is N = 72,185 of whom 3,135 carried *PCSK9* rs11591147-T (effect allele frequency 2.2%) and 59,372 carried *HMGCR* rs12916-T (effect allele frequency 57.9%).

The INTERVAL bioresource is a prospective cohort study of approximately 48,000 participants nested within a pragmatic randomized trial of blood donors (http://www.intervalstudy.org.uk).[4,5] Between 2012 and 2014, blood donors 18 years and older were consented and recruited from 25 NHSBT (National Health Service Blood and Transplant) donor centers across England. Participants are predominantly healthy individuals since people with major disease (myocardial infarction, stroke, cancer etc.) are ineligible for donation, as are those who report being unwell, or having had recent illness or infection. Participants completed online questionnaires containing basic lifestyle and health-related information, including self-reported height and weight, ethnicity, current smoking status, alcohol consumption, doctor-diagnosed anemia, use of medications (hormone replacement therapy, iron supplements) and menopausal status.

DNA was extracted from buffy coat at LGC Genomics (UK) using a Kleargene method and samples of sufficient concentration and purity were aliquoted for shipment to Affymetrix for genotyping. A modified version of the sample selection algorithm used for the UK Biobank study was implemented to ensure that samples on each plate came from participants with a mix of recruitment center, recruitment date, regional hub and gender.

NMR metabolomics was conducted by Nightingale Health Ltd (Helsinki, Finland) for EDTA plasma samples (350 µL) from 40,972 participants during 2014–2015 using the 2014 version of experimental setup and biomarker quantification algorithm. The participants were mostly non-fasted, as blood samples were taken as a routine blood donation. A few metabolites are not quantified due to the EDTA signal overlapping in the

NMR signals of glycine, glycerol, and pyruvate. In addition, glucose and lactate were omitted from the analyses due to evidence of post sample collection glycolysis progression detected in automated quality control analysis (unphysiologically low values for glucose (mean 2.9 mmol/l) and very high for lactate (mean 4.1 mmol/l)). This observation was anticipated since most of the blood samples were kept at ambient temperature for several hours prior to removal of the cells during plasma preparation, during which glucose is converted into lactate and pyruvate. In addition, concentrations of beta-hydroxybutyrate and acetoacetate were missing for most INTERVAL samples due to overlap of isopropyl alcohol in the NMR spectra. Also citrate was a priori excluded from analyses since unphysiologically high levels were observed for >20% of the samples.
The INTERVAL study was approved by the Cambridge (East) Research Ethics Committee. Informed consent was obtained from all participants.

The Avon Longitudinal Study of Parents and Children (ALSPAC) was established to understand how genetic and environmental characteristics influence health and development in parents and children (bristol.ac.uk/alspac).[6] All pregnant women resident in a defined area in the South West of England, with an expected date of delivery between 1st April 1991 and 31st December 1992, were eligible and 13,761 women were recruited. These mothers have been followed over more than two decades.[6]

NMR-based metabolomics was measured using the Nightingale 2014 version of experimental setup and biomarker quantification algorithm from EDTA plasma samples collected during the follow-up clinic assessment occurring during 2009-2011 (17-19 years after the index pregnancies). For this assessment, around 10,000 women who remained engaged with the study were invited and 50% attended. The participants attended after an overnight fast for those attending in the morning or minimum 6 hours fast for those attending in the afternoon. Metabolomics data were measured from stored EDTA plasma samples for 4,524 women. Genotype information was obtained from Illumina Human660W-Quad BeadChips run at Centre National de Génotypage, Evry, France. This study used data on genotypes of *PCSK9* rs11591147 and *HMGCR* rs12916; genotype and metabolomic data were available for 3,054 women, after exclusion of 53 on statin therapy. The study was approved by the ALSPAC Law and Ethics Committee and the UK National Health Service Research Ethics Committee and all participants provided written informed consent.

Additional recruitment of children who were potentially in the birth cohort has occurred since the children were aged 7.[7] All eligible participants have been invited to follow-up clinics with recruitment rates between 45-70%. NMR-based metabolomics was measured from EDTA plasma samples collected during follow-up at age 7, 15, and 17. *PCSK9* and *HMGCR* genotypes were obtained from 610k Illumina HumanHap arrays generated by Sample Logistics and Genotyping Facilities at the Wellcome Trust Sanger Institute, UK, and LabCorp, USA. This study utilized genotype information and metabolomics data of 4,459 children collected in follow-up at 7 years of age. The children were not required to fast for plasma sample collection. In addition, we included data of 849 children who participated in follow-up at 15 years of age, but not at age of 7, making the total N of 5,308. The study was approved by the ALSPAC Law and Ethics Committee and the UK National Health Service Research Ethics Committee. The main caregiver initially provided consent for child participation and from age 16-years the participants themselves have provided informed written consent.

China Kadoorie Biobank (CKB) Details of the China Kadoorie Biobank (CKB) design, its methods, and its participants have been reported previously (http://www.ckbiobank.org/).[8-10] Briefly, the 2004-2008 baseline survey took place in 10 (5 urban and 5 rural) localities across China, chosen from China's nationally representative Disease Surveillance Points system to retain geographic and social diversity. All 1,801,200 registered residents thought to be aged 35 to 74 years in the study areas were identified through local residential records and invited by letters and information leaflets to attend study clinics between June 2004 and July 2008. Of these registered residents, 512,869 participated, including 12,665 just outside this age range (making the actual baseline age range from 30-79 years). Because a substantial minority of registered residents would be disabled or living elsewhere, it was estimated that about one-third of the nondisabled

invitees actually living in the study areas participated. Prior to commencement of the study, ethics approval was obtained from the Oxford University Tropical Research Ethics Committee and the Chinese Center for Disease Control and Prevention Ethical Review Committee and all participants provided written informed consent. Participants provided a 10 ml nonfasting blood sample (with time since last meal recorded) that was separated into 1 buffy coat and 3 plasma aliquots. DNA extraction and genotyping was performed by BGI, Shenzhen, China. This study used data from 4,412 individuals with *HMGCR* rs12916-T genotype information available and NMR metabolomics measured from 100 µl EDTA plasma using the Nightingale Health Ltd 2016 quantification version.[11] There were no individuals with *PCSK9* rs11591147-T allele in China Kadoorie Biobank subset of 4,412 participants with NMR metabolomics data, due to the very low allele frequency of this loss-of-function variant in the Chinese population.

Northern Finland Birth Cohorts (NFBC) 1966 and 1986 were initiated to study factors affecting preterm birth and subsequent morbidity in the two northernmost provinces in Finland (www.oulu.fi/nfbc). The NFBC1986 covers 99% of all the deliveries taking place in the area during the target period (July 1985-June 1986) with the number of live born children being 9,432.[12,13] Data collection in 2001–2002 included clinical examination and serum sampling at age 15–16 for 6,621 adolescents; attendees in the 16-year field study (71%) were representative of the original cohort.[14] Metabolomics data from this time point were used for the genetic analyses in the present study. NMR metabolomics (Nightingale Health Ltd, 2016 quantification version) was measured for 5,602 serum samples, of which 95% were drawn after overnight fasting[15]. Among these, 3,216 individuals had genomic information on population stratification as well as the *PCSK9* rs11591147 and *HMGCR* rs12916 genotypes available from the CardioMetabochip. Informed written consent was obtained from all participants. The research protocols were approved by the Ethics Committee of Northern Ostrobotnia Hospital District, Finland.

The NFBC1966 included 12,058 children born into the cohort, comprising 96% of all births during 1966 in the region.[16,17] Data collection in 1997 included clinical examination and serum sampling at age 31 for 6,007 individuals. Metabolomics data from this time point were used for the genetic analyses in the present study. Attendees in the 31-year field study (52%) were representative of the original cohort.[16] NMR-based metabolomics (Nightingale Health Ltd, 2016 quantification version) was measured from 5,709 individuals with serum sample available, of which 96% were fasting samples. Among these, 4,705 individuals had genotype information *PCSK9* rs11591147 and *HMGCR* rs12196 variants based on 370k Illumina HumanHap arrays[17]. Both SNPs were imputed based on the 1000 Genome reference panel. Pregnant women were omitted from the genetic analyses. Informed written consent was obtained from all participants. The research protocols were approved by the Ethics Committee of Northern Ostrobotnia Hospital District, Finland.

FINRISK 1997 and 2007 were conducted to monitor the health of the Finnish population among persons aged 25–74 at recruitment (thl.fi/finriski).[18] In 1997, 8,444 individuals were recruited to represent the middle-aged population of five study locations across Finland. Participants completed questionnaires on use of lipid-lowering medication. Information on medication was complemented by national reimbursement records. Serum samples were stored at –70°C for later biomarker analyses. Samples were semi-fasting: participants were asked not to eat 4 hours prior to giving blood. The median fasting time was 5 h (interquartile range 4–6 h). NMR metabolomics was measured (Nightingale Health Ltd, 2016 quantification version) for 7,610 participants with serum samples available. Among these, 6,643 individuals had information on *PCSK9* rs11591147 and *HMGCR* rs12196 based on 670k Illumina HumanHap arrays after omitting of pregnant women and individuals on lipid-lowering medication. Both SNPs were imputed based on the 1000 Genome reference panel. The study was approved by the Coordinating Ethical Committee of the Helsinki and Uusimaa Hospital District, Finland.

In 2007, data was collected from altogether 7,993 individuals of whom 6,258 completed questionnaires and participated to clinical health examination. This study utilized data from a separate subcohort DILGOM

(DIetary, Lifestyle, and Genetic determinants of Obesity and Metabolic syndrome) that was formed with a specific focus on detailed phenotyping of obesity and metabolic syndrome. NMR metabolomics was measured for 4,816 participants (Nightingale Health Ltd, 2016 quantification version) from fasting serum samples. Genotypes were determined using Illumina Cardio-MetaboChip. After excluding pregnant women and individuals on lipid lowering medication, the number of individuals available with metabolomics and genotype data was 3,875. The Coordinating Ethics Committee (Institutional Review Board) of the Hospital District of Helsinki and Uusimaa has approved the FINRISK 2007 study.

Cohort Acknowledgements

INTERVAL: We thank the study co-ordination teams at the Universities of Cambridge and Oxford and at NHS Blood and Transplant, including the blood donation staff at the 25 static centers, for their help with INTERVAL participant recruitment and study fieldwork, as well as the Cambridge BioResource and NHSBT staff for their help with volunteer recruitment.

NFBC: We thank Professor Paula Rantakallio (launch of NFBC1966 and initial data collection). We gratefully acknowledge the contributions of the participants in the Northern Finland Birth Cohort 1966 study and the Northern Finland Birth Cohort 1986. We also thank all the field workers and laboratory personnel for their efforts.

ALSPAC: We are grateful to all the families who took part in this study, the midwives for their help in recruiting them, and the whole ALSPAC team, which includes interviewers, computer and laboratory technicians, clerical workers, research scientists, volunteers, managers, receptionists and nurses.

CKB: We thank the CKB participants and the project staff based at Oxford, Beijing, the 10 regional centres, and the China National CDC and its regional offices.

References

1. Shepherd J, Blauw GJ, Murphy MB, Cobbe SM, Bollen, Edward LEM, et al. The design of a prospective study of pravastatin in the elderly at risk (PROSPER). *Am J Cardiol*. 1999; 84: 1192-1197.

2. Delles C, Rankin NJ, Boachie C, McConnachie A, Ford I, Kangas A, et al. Nuclear magnetic resonance-based metabolomics identifies phenylalanine as a novel predictor of incident heart failure hospitalisation: results from PROSPER and FINRISK 1997. *Eur J Heart Fail*. 2017; doi: 10.1002/ejhf.1076.

3. Shepherd J, Blauw GJ, Murphy MB, Bollen EL, Buckley BM, Cobbe SM, et al. Pravastatin in elderly individuals at risk of vascular disease (PROSPER): a randomised controlled trial. *Lancet.* 2002; 360: 1623-1630.

4. Moore C, Sambrook J, Walker M, Tolkien Z, Kaptoge S, Allen D, et al. The INTERVAL trial to determine whether intervals between blood donations can be safely and acceptably decreased to optimise blood supply: study protocol for a randomised controlled trial. *Trials.* 2014; 15: 363.

5. Di Angelantonio E, Thompson SG, Kaptoge S, Moore C, Walker M, Armitage J, et al. Efficiency and safety of varying the frequency of whole blood donation (INTERVAL): a randomised trial of 45 000 donors. *Lancet*. 2017; 390: 2360-2371.

6. Fraser A, Macdonald-Wallis C, Tilling K, Boyd A, Golding J, Davey Smith G, et al. Cohort profile: the Avon Longitudinal Study of Parents and Children: ALSPAC mothers cohort. *Int J Epidemiol.* 2013; 42: 97-110.

7. Boyd A, Golding J, Macleod J, Lawlor DA, Fraser A, Henderson J, et al. Cohort profile: the 'children of the 90s' - the index offspring of the Avon Longitudinal Study of Parents and Children. *Int J Epidemiol.* 2013; 42: 111-127.

8. Li LM, Lv J, Guo Y, Collins R, Chen JS, Peto R, Wu F, Chen ZM; China Kadoorie Biobank (CKB) Collaborative Group. [The China Kadoorie Biobank: related methodology and baseline characteristics of the participants]. *Zhonghua Liu Xing Bing Xue Za Zhi.* 2012; 33: 249-255.

9. Chen Z, Chen J, Collins R, Guo Y, Peto R, Wu F, Li L. China Kadoorie Biobank of 0.5 million people: survey methods, baseline characteristics and long-term follow-up. *Int J Epidemiol.* 2011; 40: 1652-1666.

10. Millwood IY, Bennett DA, Holmes MV, Boxall R, Guo Y, Bian Z, et al. Association of CETP gene variants with risk for vascular and nonvascular diseases among chinese adults. *JAMA Cardiol.* 2018;3:34-43.

11. Holmes MV, Millwood IY, Kartsonaki C, Hill MR, Bennett DA, Boxall R, et al. Lipids, lipoproteins, and metabolites and risk of myocardial infarction and stroke. *J Am Coll Cardiol.* 2018; 71: 620-632.

12. Järvelin MR, Hartikainen-Sorri AL, Rantakallio P. Labour induction policy in hospitals of different levels of specialisation. *Br J Obstet Gynaecol*. 1993; 100: 310-315.

13. Kantomaa MT, Stamatakis E, Kankaanpää A, Kaakinen M, Rodriguez A, Taanila A, et al. Physical activity and obesity mediate the association between childhood motor function and adolescents' academic achievement. *Proc Natl Acad Sci U S A.* 2013; 110: 1917-1922.

14. Veltsista A, Laitinen J, Sovio U, Roma E, Järvelin M, Bakoula C. Relationship between eating behavior, breakfast consumption, and obesity among Finnish and Greek adolescents. *J Nutr Educ Behav.* 2010; 42: 417-421.

15. Würtz P, Wang Q, Kangas AJ, Richmond RC, Skarp J, Tiainen M, et al. Metabolic signatures of adiposity in young adults: Mendelian randomization analysis and effects of weight change. *PLoS Med.* 2014; 11: e1001765.

16. Järvelin M, Sovio U, King V, Lauren L, Xu B, McCarthy MI, et al. Early life factors and blood pressure at age 31 years in the 1966 northern Finland birth cohort. *Hypertension*. 2004; 44: 838-846.

17. Sabatti C, Service SK, Hartikainen AL, Pouta A, Ripatti S, Brodsky J, et al. Genome-wide association analysis of metabolic traits in a birth cohort from a founder population. *Nat Genet*. 2009; 41: 35-46.

18. Borodulin K, Tolonen H, Jousilahti P, Jula A, Juolevi A, Koskinen S, et ak. Cohort profile: the national FINRISK study. *Int J Epidemiol.* 2017; 47, 696–696i.

Table S1. Clinical characteristics of the eight cohorts included in the genetic analyses of *PCSK9* rs11591147 and *HMGCR* rs12916.

Cohort	rsID	Alleles	N	Male (%)	Age (years)	BMI (kg/m²)	Glucose (mmol/L)	Triglycerides (mmol/L)	Total cholesterol (mmol/L)	HDL cholesterol (mmol/L)	Friedewald LDL cholesterol (mmol/L)
**INTERVAL** (N=40,972)	rs11591147	G/G	39554	49.50	43.5 ± 14.3	26.4 ± 4.6	*	1.15 ± 0.51	4.22 ± 1.00	1.43 ± 0.36	2.26
		G/T	1395	50.40	44.1 ± 14.3	26.5 ± 4.7	*	1.13 ± 0.52	3.87 ± 0.96	1.43 ± 0.37	1.93
		T/T	No rs11591147 T/T homozygotes in INTERVAL.								
	rs12916	C/C	6533	48.70	43.5 ± 14.2	26.3 ± 4.7	*	1.16 ± 0.50	4.28 ± 1.02	1.44 ± 0.37	2.32
		C/T	19809	49.70	45.5 ± 14.2	26.3 ± 4.6	*	1.15 ± 0.51	4.22 ± 1.00	1.44 ± 0.36	2.26
		T/T	14580	49.60	45.5 ± 14.3	26.4 ± 4.7	*	1.15 ± 0.51	4.15 ± 1.00	1.43 ± 0.37	2.20
**ALSPAC kids** (N=5,308)	rs11591147	G/G	5118	50.31	8.8 ± 2.9	17.1 ± 3.1	5.2 ± 0.4	1.03 ± 0.50	4.31 ± 0.70	1.48 ± 0.31	2.36 ± 0.59
		G/T	188	56.38	8.9 ± 3.0	17.3 ± 3.4	5.3 ± 0.4	0.98 ± 0.49	4.08 ± 0.72	1.53 ± 0.34	2.10 ± 0.58
		T/T	2	50.00	7.7 ± 0.1	19.3 ± 4.7	*no data*	1.46 ± 0.72	3.69 ± 0.25	1.25 ± 0.42	1.77 ± 0.35
	rs12916	C/C	845	49.47	8.8 ± 3.0	17.1 ± 3.2	5.3 ± 0.4	1.06 ± 0.53	4.38 ± 0.71	1.48 ± 0.33	2.41 ± 0.60
		C/T	2605	51.17	8.8 ± 2.9	17.1 ± 3.1	5.2 ± 0.4	1.02 ± 0.49	4.30 ± 0.70	1.48 ± 0.31	2.36 ± 0.60
		T/T	1858	50.11	8.8 ± 2.9	17.1 ± 3.1	5.2 ± 0.4	1.03 ± 0.51	4.26 ± 0.70	1.49 ± 0.31	2.30 ± 0.59
**ALSPAC moms** (N=3,054)	rs11591147	G/G	2929	0	NA	NA	5.2 ± 0.8	1.01 ± 0.57	4.93 ± 0.85	1.49 ± 0.38	2.99 ± 0.79
		G/T	125	0	NA	NA	5.4 ± 1.4	1.03 ± 0.65	4.71 ± 0.82	1.51 ± 0.44	2.75 ± 0.77
		T/T	No rs11591147 T/T homozygotes in ALSPAC moms.								
	rs12916	C/C	503	0	NA	NA	5.3 ± 1.2	1.02 ± 0.55	4.96 ± 0.87	1.48 ± 0.36	3.02 ± 0.81
		C/T	1488	0	NA	NA	5.2 ± 0.6	1.02 ± 0.58	4.97 ± 0.84	1.49 ± 0.39	3.02 ± 0.79
		T/T	1063	0	NA	NA	5.3 ± 0.9	1.00 ± 0.56	4.85 ± 0.86	1.50 ± 0.39	2.90 ± 0.79
**China Kadoorie Biobank** (N=4,412)	rs11591147	G/G	No individuals with rs11591147-T allele in the China Kadoorie Biobank subset.								
		G/T									
		T/T									
	rs12916	C/C	1203	50.71	45.8	23.8 ± 3.6	6.1 ± 2.9	2.07 ± 1.84	4.65 ± 1.00	1.21 ± 0.29	2.50 ± 0.89
		C/T	2241	50.56	46.5	24.0 ± 3.6	6.1 ± 2.8	2.08 ± 1.87	4.62 ± 1.02	1.21 ± 0.29	2.46 ± 0.93
		T/T	969	46.03	46.3	24.0 ± 3.5	6.0 ± 2.4	2.01 ± 1.48	4.47 ± 0.94	1.2 ± 0.29	2.36 ± 0.85

Values are mean ± SD.

* Omitted due to severe glycolysis progression post-sample collection.

Table S1 (Continued)

Cohort	rsID	Alleles	N	Male (%)	Age (years)	BMI (kg/m²)	Glucose (mmol/L)	Triglycerides (mmol/L)	Total cholesterol (mmol/L)	HDL cholesterol (mmol/L)	Friedewald LDL cholesterol (mmol/L)
**NFBC 1966** (N=4,705)	rs11591147	G/G	4402	48.98	31.2 ± 0.4	24.6 ± 4.1	5.0 ± 0.6	1.16 ± 0.69	5.08 ± 0.97	1.55 ± 0.38	3.00 ± 0.90
		G/T	300	53.33	31.1 ± 0.4	24.5 ± 4.1	5.0 ± 0.4	1.18 ± 0.70	4.62 ± 0.86	1.53 ± 0.37	2.54 ± 0.79
		T/T	3	100.00	31.4 ± 0.2	23.1 ± 0.3	4.9 ± 0.1	1.02 ± 0.49	4.11 ± 0.39	1.39 ± 0.22	2.25 ± 0.23
	rs12916	C/C	886	49.32	31.1 ± 0.4	24.6 ± 4.2	5.1 ± 0.7	1.21 ± 0.79	5.12 ± 1.00	1.55 ± 0.38	3.03 ± 0.91
		C/T	2329	49.03	31.2 ± 0.4	24.6 ± 4.1	5.0 ± 0.5	1.15 ± 0.67	5.07 ± 0.96	1.54 ± 0.37	3.00 ± 0.89
		T/T	1490	49.66	31.2 ± 0.4	24.6 ± 4.1	5.0 ± 0.5	1.15 ± 0.65	4.99 ± 0.97	1.56 ± 0.38	2.90 ± 0.90
**NFBC 1986** (N=3,216**)**	rs11591147	G/G	2975	49.31	16.1 ± 0.4	21.3 ± 3.5	5.2 ± 0.8	0.86 ± 0.45	4.30 ± 0.80	1.40 ± 0.30	2.51 ± 0.68
		G/T	233	44.64	16.1 ± 0.4	21.4 ± 3.4	5.3 ± 1.3	0.83 ± 0.35	3.90 ± 0.73	1.41 ± 0.28	2.11 ± 0.62
		T/T	8	25.00	16.1 ± 0.3	20.0 ± 2.2	4.8 ± 0.6	0.64 ± 0.22	3.80 ± 0.53	1.63 ± 0.38	1.88 ± 0.34
	rs12916	C/C	625	48.80	16.1 ± 0.4	21.4 ± 3.6	5.2 ± 1.2	0.88 ± 0.44	4.33 ± 0.77	1.39 ± 0.31	2.53 ± 0.67
		C/T	1526	48.95	16.1 ± 0.4	21.3 ± 3.6	5.2 ± 0.6	0.85 ± 0.45	4.30 ± 0.82	1.40 ± 0.30	2.51 ± 0.71
		T/T	1065	48.92	16.1 ± 0.4	21.2 ± 3.2	5.2 ± 0.8	0.84 ± 0.43	4.19 ± 0.77	1.41 ± 0.29	2.40 ± 0.65
**FINRISK 1997** (N=6,643)	rs11591147	G/G	6103	48.30	47.7 ± 13.0	26.6 ± 4.5	4.6 ± 1.0 **	1.48 ± 1.03	5.57 ± 1.05	1.40 ± 0.36	3.51 ± 0.92
		G/T	523	47.42	48.4 ± 13.2	26.4 ± 4.3	4.6 ± 1.2 **	1.40 ± 0.92	5.16 ± 0.96	1.42 ± 0.36	3.10 ± 0.84
		T/T	17	52.94	50.6 ± 15.5	26.6 ± 4.8	4.5 ± 0.3 **	1.84 ± 1.11	4.73 ± 0.96	1.41 ± 0.43	2.52 ± 0.85
	rs12916	C/C	1376	47.82	48.1 ± 13.3	26.7 ± 4.6	4.6 ± 1.1 **	1.46 ± 0.92	5.59 ± 1.06	1.39 ± 0.35	3.55 ± 0.92
		C/T	3295	47.98	47.7 ± 12.8	26.6 ± 4.5	4.6 ± 1.1 **	1.49 ± 1.09	5.56 ± 1.04	1.40 ± 0.36	3.50 ± 0.92
		T/T	1972	48.99	47.7 ± 13.2	26.5 ± 4.5	4.6 ± 1.0 **	1.44 ± 0.98	5.44 ± 1.06	1.41 ± 0.36	3.39 ± 0.93
**FINRISK 2007** (N=3,875)	rs11591147	G/G	3534	44.99	50.3 ± 13.4	26.9 ± 4.8	5.9 ± 0.9	1.37 ± 0.89	5.40 ± 0.98	1.45 ± 0.37	3.33 ± 0.85
		G/T	334	47.01	50.8 ± 13.3	27.1 ± 4.6	5.9 ± 0.9	1.46 ± 1.03	5.00 ± 0.92	1.46 ± 0.38	2.89 ± 0.76
		T/T	7	71.43	50.9 ± 13.8	27.2 ± 6.4	5.9 ± 0.7	1.20 ± 0.82	4.34 ± 0.74	1.58 ± 0.42	2.22 ± 0.61
	rs12916	C/C	793	44.39	49.8 ± 13.3	27.1 ± 4.9	5.9 ± 0.7	1.38 ± 0.83	5.47 ± 0.99	1.45 ± 0.38	3.40 ± 0.88
		C/T	1916	47.13	50.2 ± 13.5	26.8 ± 4.8	5.8 ± 0.8	1.39 ± 0.88	5.36 ± 0.99	1.45 ± 0.37	3.28 ± 0.85
		T/T	1166	42.62	51.0 ± 13.3	26.9 ± 4.8	5.9 ± 1.0	1.37 ± 0.99	5.30 ± 0.97	1.46 ± 0.38	3.23 ± 0.83

** Glucose based on NMR, measured from standard serum tubes 15 years after blood sampling.

Table S2. Metabolite mean (SD) concentrations in PROSPER and cohorts used for genetic analyses.

Metabolic measure	PROSPER total N (on statin)	PROSPER total mean (SD)	Pravastatin mean (SD)	Placebo mean (SD)	Eight cohorts N	Eight cohorts mean (SD)
**Lipoprotein subclass particle concentrations (P)**						
Extremely large VLDL (µmol/L)	5346 (2647)	0.00012 (0.00012)	0.000092 (0.00011)	0.000155 (0.00012)	72185	0.00014 (0.00013)
Very large VLDL (µmol/L)	5339 (2642)	0.00066 (0.00068)	0.000528 (0.00062)	0.000785 (0.00072)	72185	0.00059 (0.00065)
Large VLDL (µmol/L)	5349 (2650)	0.00461 (0.00375)	0.00395 (0.00345)	0.00526 (0.00391)	72184	0.00347 (0.00319)
Medium VLDL (µmol/L)	5358 (2658)	0.0173 (0.00944)	0.0153 (0.00854)	0.0193 (0.00984)	72185	0.0139 (0.0080)
Small VLDL (µmol/L)	5359 (2659)	0.0343 (0.0111)	0.0308 (0.00959)	0.0377 (0.0114)	72185	0.0286 (0.0105)
Very small VLDL (µmol/L)	5359 (2659)	0.0439 (0.0104)	0.0385 (0.00783)	0.0493 (0.0097)	72185	0.0404 (0.0111)
IDL (µmol/L)	5359 (2659)	0.101 (0.0265)	0.0847 (0.0195)	0.116 (0.0229)	72166	0.105 (0.029)
Large LDL (µmol/L)	5359 (2659)	0.155 (0.0468)	0.127 (0.0348)	0.183 (0.0404)	72185	0.164 (0.049)
Medium LDL (µmol/L)	5359 (2659)	0.122 (0.0401)	0.0984 (0.0301)	0.145 (0.0347)	72185	0.126 (0.042)
Small LDL (µmol/L)	5359 (2659)	0.143 (0.0425)	0.118 (0.0326)	0.167 (0.0369)	72185	0.148 (0.046)
Very large HDL (µmol/L)	5359 (2659)	0.407 (0.171)	0.394 (0.179)	0.419 (0.162)	72185	0.530 (0.210)
Large HDL (µmol/L)	5359 (2659)	1.05 (0.411)	1.09 (0.416)	1.00 (0.401)	72185	1.28 (0.56)
Medium HDL (µmol/L)	5359 (2659)	1.78 (0.292)	1.81 (0.297)	1.76 (0.285)	72185	1.89 (0.48)
Small HDL (µmol/L)	5359 (2659)	4.42 (0.389)	4.48 (0.401)	4.37 (0.369)	72185	4.64 (0.67)
**Cholesterol (C) in subfractions and lipoprotein subclasses**						
Total C (mmol/L)	5359 (2659)	4.197 (0.946)	3.656 (0.720)	4.731 (0.832)	72166	4.4 (0.978)
Remnant C (mmol/L)	5359 (2659)	1.479 (0.426)	1.237 (0.318)	1.718 (0.382)	72166	1.4 (0.433)
VLDL C (mmol/L)	5359 (2659)	0.840 (0.276)	0.713 (0.220)	0.965 (0.267)	72165	0.727 (0.272)
Extremely large VLDL (mmol/L)	5346 (2647)	0.006 (0.005)	0.004 (0.004)	0.007 (0.005)	72185	0.0057 (0.0052)
Very large VLDL (mmol/L)	5339 (2642)	0.016 (0.014)	0.012 (0.012)	0.019 (0.015)	72182	0.015 (0.015)
Large VLDL (mmol/L)	5349 (2650)	0.066 (0.052)	0.053 (0.046)	0.078 (0.054)	72183	0.054 (0.048)
Medium VLDL (mmol/L)	5358 (2658)	0.174 (0.089)	0.143 (0.076)	0.205 (0.091)	72185	0.144 (0.077)
Small VLDL (mmol/L)	5359 (2659)	0.287 (0.083)	0.246 (0.065)	0.327 (0.078)	72185	0.231 (0.083)
Very small VLDL (mmol/L)	5359 (2659)	0.292 (0.063)	0.256 (0.047)	0.328 (0.057)	72184	0.276 (0.074)
IDL (mmol/L)	5359 (2659)	0.639 (0.187)	0.524 (0.135)	0.752 (0.160)	72185	0.680 (0.199)
LDL C (mmol/L)	5359 (2659)	1.385 (0.490)	1.091 (0.362)	1.674 (0.423)	72165	1.5 (0.510)
Large LDL (mmol/L)	5359 (2659)	0.725 (0.248)	0.575 (0.182)	0.873 (0.213)	72185	0.791 (0.258)
Medium LDL (mmol/L)	5359 (2659)	0.407 (0.153)	0.315 (0.113)	0.497 (0.133)	72185	0.427 (0.160)

Small LDL (mmol/L)	5359 (2659)	0.253 (0.090)	0.200 (0.068)	0.305 (0.078)	72185	0.261 (0.096)
HDL C (mmol/L)	5359 (2659)	1.334 (0.274)	1.329 (0.277)	1.340 (0.271)	72166	1.5 (0.352)
HDL2 C (mmol/L)	5359 (2659)	0.866 (0.264)	0.894 (0.266)	0.839 (0.258)	72162	0.953 (0.325)
HDL3 C (mmol/L)	5359 (2659)	0.468 (0.073)	0.435 (0.050)	0.501 (0.078)	72162	0.518 (0.043)
Very large HDL (mmol/L)	5359 (2659)	0.238 (0.083)	0.227 (0.086)	0.248 (0.080)	72185	0.278 (0.102)
Large HDL (mmol/L)	5359 (2659)	0.325 (0.141)	0.341 (0.141)	0.309 (0.139)	72185	0.391 (0.184)
Medium HDL (mmol/L)	5359 (2659)	0.369 (0.067)	0.375 (0.066)	0.364 (0.066)	72184	0.394 (0.114)
Small HDL (mmol/L)	5359 (2659)	0.401 (0.050)	0.385 (0.045)	0.418 (0.049)	72184	0.406 (0.075)
**Cholesteryl esters (CE)**						
Esterified C (mmol/L)	5331 (2636)	2.994 (0.680)	2.618 (0.525)	3.362 (0.609)	71673	3.1 (0.716)
Extremely large VLDL (mmol/L)	5346 (2647)	0.003 (0.003)	0.002 (0.002)	0.004 (0.003)	72185	0.0035 (0.0031)
Very large VLDL (mmol/L)	5339 (2642)	0.009 (0.008)	0.007 (0.007)	0.011 (0.008)	72180	0.0082 (0.0078)
Large VLDL (mmol/L)	5349 (2650)	0.037 (0.027)	0.029 (0.023)	0.044 (0.028)	72178	0.032 (0.025)
Medium VLDL (mmol/L)	5358 (2658)	0.103 (0.051)	0.081 (0.041)	0.124 (0.051)	72182	0.090 (0.044)
Small VLDL (mmol/L)	5359 (2659)	0.180 (0.053)	0.152 (0.041)	0.209 (0.049)	72182	0.150 (0.056)
Very small VLDL (mmol/L)	5359 (2659)	0.196 (0.041)	0.172 (0.031)	0.219 (0.036)	72183	0.193 (0.053)
IDL (mmol/L)	5359 (2659)	0.450 (0.129)	0.369 (0.094)	0.529 (0.109)	72185	0.489 (0.143)
Large LDL (mmol/L)	5359 (2659)	0.505 (0.189)	0.391 (0.139)	0.617 (0.162)	72180	0.560 (0.196)
Medium LDL (mmol/L)	5359 (2659)	0.276 (0.126)	0.201 (0.093)	0.350 (0.109)	72181	0.295 (0.129)
Small LDL (mmol/L)	5359 (2659)	0.170 (0.074)	0.126 (0.055)	0.213 (0.065)	72181	0.179 (0.078)
Very large HDL (mmol/L)	5359 (2659)	0.183 (0.060)	0.175 (0.061)	0.190 (0.058)	72185	0.211 (0.074)
Large HDL (mmol/L)	5359 (2659)	0.251 (0.105)	0.263 (0.106)	0.238 (0.104)	72185	0.308 (0.140)
Medium HDL (mmol/L)	5359 (2659)	0.291 (0.052)	0.298 (0.052)	0.284 (0.051)	72183	0.320 (0.090)
Small HDL (mmol/L)	5359 (2659)	0.282 (0.049)	0.262 (0.041)	0.303 (0.047)	72175	0.294 (0.066)
**Free cholesterol (FC)**						
Free C (mmol/L)	5333 (2637)	1.207 (0.285)	1.040 (0.217)	1.370 (0.246)	71665	1.2 (0.273)
Extremely large VLDL (mmol/L)	5346 (2647)	0.002 (0.002)	0.002 (0.002)	0.003 (0.002)	72185	0.0022 (0.0023)
Very large VLDL (mmol/L)	5339 (2642)	0.007 (0.007)	0.005 (0.006)	0.008 (0.007)	72185	0.0066 (0.0069)
Large VLDL (mmol/L)	5349 (2650)	0.029 (0.025)	0.024 (0.023)	0.034 (0.026)	72185	0.022 (0.023)
Medium VLDL (mmol/L)	5358 (2658)	0.071 (0.040)	0.061 (0.036)	0.081 (0.041)	72185	0.055 (0.035)
Small VLDL (mmol/L)	5359 (2659)	0.106 (0.032)	0.094 (0.027)	0.119 (0.032)	72185	0.081 (0.029)
Very small VLDL (mmol/L)	5359 (2659)	0.097 (0.024)	0.084 (0.018)	0.109 (0.022)	72182	0.083 (0.023)
IDL (mmol/L)	5359 (2659)	0.189 (0.059)	0.154 (0.043)	0.224 (0.053)	72183	0.191 (0.057)
Large LDL (mmol/L)	5359 (2659)	0.220 (0.060)	0.184 (0.044)	0.255 (0.052)	72185	0.231 (0.063)

Medium LDL (mmol/L)	5359 (2659)	0.131 (0.028)	0.114 (0.021)	0.147 (0.024)	72185	0.132 (0.031)
Small LDL (mmol/L)	5359 (2659)	0.083 (0.016)	0.074 (0.013)	0.092 (0.014)	72185	0.082 (0.019)
Very large HDL (mmol/L)	5359 (2659)	0.055 (0.025)	0.052 (0.026)	0.058 (0.024)	72185	0.068 (0.029)
Large HDL (mmol/L)	5359 (2659)	0.074 (0.036)	0.077 (0.036)	0.071 (0.036)	72185	0.083 (0.044)
Medium HDL (mmol/L)	5359 (2659)	0.078 (0.017)	0.077 (0.016)	0.079 (0.018)	72185	0.074 (0.024)
Small HDL (mmol/L)	5359 (2659)	0.119 (0.014)	0.123 (0.014)	0.115 (0.013)	72185	0.112 (0.018)
**Triglycerides (TG) in major subfractions and lipoprotein subclasses**						
Total TG (mmol/L)	5359 (2659)	1.307 (0.575)	1.179 (0.513)	1.432 (0.605)	72166	1.2 (0.530)
VLDL TG (mmol/L)	5359 (2659)	0.874 (0.485)	0.788 (0.444)	0.960 (0.508)	72166	0.715 (0.419)
Extremely large VLDL (mmol/L)	5346 (2647)	0.018 (0.018)	0.013 (0.016)	0.022 (0.018)	72175	0.021 (0.020)
Very large VLDL (mmol/L)	5339 (2642)	0.039 (0.042)	0.032 (0.039)	0.046 (0.044)	72176	0.034 (0.039)
Large VLDL (mmol/L)	5349 (2650)	0.157 (0.128)	0.137 (0.119)	0.176 (0.133)	72182	0.113 (0.107)
Medium VLDL (mmol/L)	5358 (2658)	0.298 (0.171)	0.271 (0.158)	0.324 (0.179)	72185	0.231 (0.143)
Small VLDL (mmol/L)	5359 (2659)	0.245 (0.101)	0.226 (0.091)	0.263 (0.107)	72185	0.209 (0.091)
Very small VLDL (mmol/L)	5359 (2659)	0.118 (0.038)	0.108 (0.033)	0.128 (0.041)	72185	0.104 (0.037)
IDL (mmol/L)	5359 (2659)	0.119 (0.035)	0.108 (0.028)	0.129 (0.038)	72185	0.111 (0.040)
LDL TG (mmol/L)	5359 (2659)	0.174 (0.058)	0.154 (0.048)	0.193 (0.061)	72166	0.174 (0.072)
Large LDL (mmol/L)	5359 (2659)	0.101 (0.032)	0.090 (0.026)	0.111 (0.033)	72185	0.099 (0.038)
Medium LDL (mmol/L)	5359 (2659)	0.045 (0.017)	0.039 (0.014)	0.050 (0.017)	72184	0.046 (0.021)
Small LDL (mmol/L)	5359 (2659)	0.028 (0.011)	0.024 (0.009)	0.031 (0.012)	72185	0.029 (0.013)
HDL TG (mmol/L)	5359 (2659)	0.140 (0.041)	0.129 (0.035)	0.150 (0.043)	72166	0.150 (0.046)
Very large HDL (mmol/L)	5359 (2659)	0.017 (0.007)	0.014 (0.007)	0.019 (0.007)	72185	0.019 (0.0086)
Large HDL (mmol/L)	5359 (2659)	0.031 (0.012)	0.029 (0.011)	0.034 (0.013)	72184	0.037 (0.017)
Medium HDL (mmol/L)	5359 (2659)	0.041 (0.015)	0.038 (0.013)	0.045 (0.015)	72185	0.044 (0.015)
Small HDL (mmol/L)	5359 (2659)	0.050 (0.015)	0.048 (0.014)	0.052 (0.016)	72184	0.050 (0.017)
**Phospholipids (PL) in lipoprotein subclasses**						
Extremely large VLDL (mmol/L)	5346 (2647)	0.003 (0.003)	0.002 (0.003)	0.004 (0.003)	72185	0.0034 (0.0035)
Very large VLDL (mmol/L)	5339 (2642)	0.010 (0.011)	0.008 (0.010)	0.012 (0.012)	72185	0.0100 (0.011)
Large VLDL (mmol/L)	5349 (2650)	0.045 (0.039)	0.038 (0.036)	0.052 (0.041)	72180	0.037 (0.034)
Medium VLDL (mmol/L)	5358 (2658)	0.112 (0.061)	0.098 (0.054)	0.126 (0.064)	72185	0.098 (0.053)
Small VLDL (mmol/L)	5359 (2659)	0.157 (0.045)	0.143 (0.039)	0.170 (0.047)	72185	0.134 (0.044)
Very small VLDL (mmol/L)	5359 (2659)	0.152 (0.044)	0.128 (0.032)	0.176 (0.040)	72185	0.146 (0.043)
IDL (mmol/L)	5359 (2659)	0.260 (0.068)	0.220 (0.051)	0.301 (0.059)	72185	0.286 (0.075)
Large LDL (mmol/L)	5359 (2659)	0.279 (0.068)	0.238 (0.050)	0.319 (0.058)	72185	0.302 (0.074)

Medium LDL (mmol/L)	5359 (2659)	0.173 (0.039)	0.151 (0.030)	0.196 (0.034)	72185	0.185 (0.045)
Small LDL (mmol/L)	5359 (2659)	0.124 (0.024)	0.111 (0.020)	0.137 (0.021)	72185	0.134 (0.029)
Very large HDL (mmol/L)	5359 (2659)	0.163 (0.094)	0.163 (0.098)	0.163 (0.090)	72184	0.254 (0.116)
Large HDL (mmol/L)	5359 (2659)	0.305 (0.120)	0.319 (0.121)	0.290 (0.117)	72185	0.385 (0.157)
Medium HDL (mmol/L)	5359 (2659)	0.349 (0.061)	0.357 (0.061)	0.341 (0.059)	72185	0.388 (0.089)
Small HDL (mmol/L)	5359 (2659)	0.535 (0.074)	0.566 (0.067)	0.505 (0.067)	72185	0.581 (0.088)
Cholines (mmol/L)	5333 (2637)	2.186 (0.347)	2.058 (0.316)	2.310 (0.330)	71664	2.3 (0.432)
Phosphatidylcholine (mmol/L)	5332 (2636)	1.799 (0.303)	1.698 (0.277)	1.899 (0.295)	71279	1.9 (0.394)
Sphingomyelins (mmol/L)	5303 (2622)	0.472 (0.097)	0.436 (0.084)	0.507 (0.097)	63437	0.440 (0.090)
Phosphoglycerides (mmol/L)	5330 (2635)	1.780 (0.321)	1.675 (0.294)	1.882 (0.313)	71666	1.9 (0.410)
Triglycerides to phosphoglycerides (ratio)	5330 (2635)	0.669 (0.251)	0.658 (0.247)	0.679 (0.255)	71658	0.590 (0.255)
**Total lipid concentrations (L)**						
Extremely large VLDL (mmol/L)	5346 (2647)	0.027 (0.026)	0.020 (0.023)	0.033 (0.027)	72185	0.030 (0.028)
Very large VLDL (mmol/L)	5339 (2642)	0.065 (0.067)	0.052 (0.061)	0.078 (0.070)	72185	0.059 (0.064)
Large VLDL (mmol/L)	5349 (2650)	0.268 (0.218)	0.228 (0.200)	0.307 (0.227)	72184	0.204 (0.188)
Medium VLDL (mmol/L)	5358 (2658)	0.584 (0.315)	0.512 (0.284)	0.655 (0.329)	72185	0.473 (0.269)
Small VLDL (mmol/L)	5359 (2659)	0.688 (0.216)	0.615 (0.184)	0.760 (0.220)	72185	0.575 (0.207)
Very small VLDL (mmol/L)	5359 (2659)	0.563 (0.131)	0.492 (0.098)	0.633 (0.122)	72185	0.526 (0.142)
IDL (mmol/L)	5359 (2659)	1.018 (0.275)	0.851 (0.201)	1.183 (0.236)	72166	1.1 (0.296)
Large LDL (mmol/L)	5359 (2659)	1.105 (0.335)	0.904 (0.248)	1.304 (0.289)	72185	1.2 (0.355)
Medium LDL (mmol/L)	5359 (2659)	0.625 (0.202)	0.505 (0.151)	0.743 (0.175)	72185	0.658 (0.215)
Small LDL (mmol/L)	5359 (2659)	0.405 (0.119)	0.336 (0.091)	0.473 (0.103)	72185	0.423 (0.131)
Very large HDL (mmol/L)	5359 (2659)	0.417 (0.175)	0.404 (0.182)	0.430 (0.166)	72185	0.551 (0.216)
Large HDL (mmol/L)	5359 (2659)	0.660 (0.264)	0.689 (0.267)	0.633 (0.259)	72185	0.813 (0.350)
Medium HDL (mmol/L)	5359 (2659)	0.759 (0.126)	0.769 (0.128)	0.749 (0.123)	72185	0.825 (0.204)
Small HDL (mmol/L)	5359 (2659)	0.986 (0.086)	0.998 (0.089)	0.975 (0.082)	72185	1.0 (0.146)
**Lipoprotein particle size**						
VLDL particle size (nm)	5359 (2659)	36 (1.2)	36 (1.3)	36 (1.1)	72166	36.1 (1.2)
LDL particle size (nm)	5359 (2659)	24 (0.1)	24 (0.1)	24 (0.1)	72166	23.7 (0.148)
HDL particle size (nm)	5359 (2659)	10 (0.2)	10 (0.2)	10 (0.2)	72166	10.1 (0.248)
**Apolipoproteins**						
Apolipoprotein B (g/L)	5359 (2659)	0.928 (0.219)	0.807 (0.168)	1.046 (0.198)	72157	0.834 (0.202)
Apolipoprotein A-I (g/L)	5359 (2659)	1.549 (0.161)	1.529 (0.166)	1.569 (0.154)	72164	1.5 (0.220)

ApoB to ApoA-I (ratio)	5359 (2659)	0.602 (0.140)	0.533 (0.114)	0.671 (0.129)	72157	0.555 (0.140)
**Fatty acids (FA)**						
Total fatty acids (mmol/L)	5333 (2637)	10.882 (2.283)	9.968 (1.912)	11.777 (2.262)	71627	11.0 (2.5)
Saturated fatty acids (mmol/L)	5331 (2636)	4.223 (0.957)	3.890 (0.835)	4.549 (0.956)	71534	4.1 (0.991)
MUFA (mmol/L)	5333 (2637)	2.762 (0.837)	2.536 (0.751)	2.983 (0.858)	71538	2.9 (0.897)
PUFA (mmol/L)	5332 (2637)	3.895 (0.781)	3.542 (0.632)	4.242 (0.758)	71627	4.0 (0.832)
Omega-6 (mmol/L)	5331 (2636)	3.464 (0.722)	3.141 (0.587)	3.781 (0.701)	71627	3.6 (0.733)
Linoleic acid (mmol/L)	5332 (2637)	2.783 (0.664)	2.472 (0.529)	3.088 (0.641)	71627	2.9 (0.624)
Omega-3 (mmol/L)	5333 (2637)	0.431 (0.130)	0.401 (0.114)	0.461 (0.137)	71629	0.438 (0.144)
Docosahexaenoic acid (mmol/L)	5333 (2637)	0.152 (0.053)	0.143 (0.047)	0.161 (0.056)	71631	0.147 (0.056)
**Fatty acid ratios**						
Saturated fatty acids (%)	5331 (2636)	38.8 (3.1)	39.0 (3.3)	38.6 (2.8)	71532	37.3 (1.9)
MUFA (%)	5333 (2637)	25.1 (3.9)	25.2 (4.1)	25.1 (3.8)	71535	25.7 (3.1)
PUFA (%)	5332 (2637)	36.1 (4.0)	35.9 (4.1)	36.3 (3.9)	71624	37.1 (3.3)
Omega-6 (%)	5331 (2636)	32.1 (3.9)	31.8 (4.0)	32.4 (3.9)	71624	33.1 (3.1)
Linoleic acid (%)	5332 (2637)	25.7 (4.0)	25.0 (3.9)	26.4 (3.9)	71624	26.8 (3.1)
Omega-3 (%)	5333 (2637)	4.0 (1.1)	4.1 (1.1)	3.9 (1.0)	71625	4.0 (0.946)
Docosahexaenoic acid (%)	5333 (2637)	1.4 (0.4)	1.4 (0.5)	1.4 (0.4)	71627	1.3 (0.406)
Degree of unsaturation	5332 (2637)	1.209 (0.1)	1.2 (0.1)	1.2 (0.1)	71624	1.2 (0.069)
**Total cholesterol to total lipids ratio (C%)**						
Extremely large VLDL (%)	5346 (2647)	23.2 (10.1)	23.3 (12.7)	23.1 (6.8)	67184	18.4 (4.8)
Very large VLDL (%)	5339 (2642)	27.0 (13.6)	25.4 (14.8)	28.5 (12.2)	63076	27.8 (9.8)
Large VLDL (%)	5349 (2650)	25.1 (7.0)	23.1 (7.1)	27.0 (6.3)	65516	27.3 (5.8)
Medium VLDL (%)	5358 (2658)	30.4 (5.2)	28.5 (4.9)	32.3 (4.6)	72036	31.1 (5.0)
Small VLDL (%)	5359 (2659)	42.3 (5.3)	40.6 (5.0)	43.9 (5.1)	72124	40.4 (5.3)
Very small VLDL (%)	5359 (2659)	52.3 (3.9)	52.3 (4.0)	52.2 (3.7)	72146	52.4 (3.9)
IDL (%)	5359 (2659)	62.4 (2.7)	61.3 (2.6)	63.5 (2.4)	72131	62.7 (3.0)
Large LDL (%)	5359 (2659)	64.8 (3.6)	63.0 (3.5)	66.6 (2.5)	72114	65.7 (3.2)
Medium LDL (%)	5359 (2659)	63.8 (5.2)	61.2 (5.4)	66.4 (3.5)	71883	63.7 (5.1)
Small LDL (%)	5359 (2659)	61.2 (5.6)	58.5 (5.7)	63.9 (4.0)	71891	60.4 (5.5)
Very large HDL (%)	5359 (2659)	58.2 (9.2)	57.4 (10.1)	58.9 (8.1)	71726	50.9 (5.9)
Large HDL (%)	5359 (2659)	47.9 (6.4)	48.5 (5.6)	47.3 (7.0)	71092	47.3 (3.9)
Medium HDL (%)	5359 (2659)	48.6 (2.7)	48.7 (2.4)	48.5 (3.1)	71985	47.1 (3.9)

Small HDL (%)	5359 (2659)	40.8 (4.6)	38.6 (3.7)	42.9 (4.3)	72133	39.0 (4.1)
**Triglycerides to total lipids ratio (TG%)**						
Extremely large VLDL (%)	5346 (2647)	61.4 (15.5)	57.9 (19.7)	64.9 (8.3)	67187	70.9 (5.5)
Very large VLDL (%)	5339 (2642)	53.8 (17.8)	53.7 (20.9)	53.8 (14.1)	63030	56.2 (9.9)
Large VLDL (%)	5349 (2650)	58.3 (9.2)	60.1 (10.7)	56.4 (7.0)	65500	55.2 (36.4)
Medium VLDL (%)	5358 (2658)	50.3 (5.4)	52.3 (5.2)	48.4 (4.9)	72018	48.0 (5.5)
Small VLDL (%)	5359 (2659)	34.8 (5.2)	35.9 (5.1)	33.6 (5.1)	72121	36.0 (5.2)
Very small VLDL (%)	5359 (2659)	21.0 (4.4)	21.9 (4.5)	20.1 (4.2)	72154	20.0 (4.4)
IDL (%)	5359 (2659)	12.0 (3.0)	12.9 (2.9)	11.1 (2.7)	72152	10.6 (2.9)
Large LDL (%)	5359 (2659)	9.4 (2.5)	10.2 (2.5)	8.7 (2.2)	72138	8.5 (2.5)
Medium LDL (%)	5359 (2659)	7.4 (2.1)	7.9 (2.2)	6.8 (1.9)	71882	7.1 (2.4)
Small LDL (%)	5359 (2659)	7.0 (2.2)	7.3 (2.2)	6.6 (2.1)	71891	6.8 (2.4)
Very large HDL (%)	5359 (2659)	4.5 (3.1)	4.0 (3.2)	5.0 (2.9)	71730	3.7 (2.3)
Large HDL (%)	5359 (2659)	5.2 (2.7)	4.5 (2.0)	5.9 (3.0)	71096	4.9 (2.2)
Medium HDL (%)	5359 (2659)	5.5 (2.0)	5.0 (1.7)	6.1 (2.2)	71995	5.6 (2.3)
Small HDL (%)	5359 (2659)	5.1 (1.5)	4.8 (1.3)	5.4 (1.6)	72133	4.9 (1.6)
**Phospholipids to total lipids ratio (PL%)**						
Extremely large VLDL (%)	5346 (2647)	11.6 (4.2)	11.8 (5.4)	11.3 (2.2)	67180	10.8 (2.7)
Very large VLDL (%)	5339 (2642)	12.8 (5.3)	11.1 (5.7)	14.5 (4.3)	63096	16.1 (4.2)
Large VLDL (%)	5349 (2650)	15.5 (3.4)	14.9 (3.7)	16.1 (3.0)	65520	17.8 (2.4)
Medium VLDL (%)	5358 (2658)	19.3 (1.2)	19.2 (1.1)	19.3 (1.3)	72034	20.9 (1.0)
Small VLDL (%)	5359 (2659)	23.0 (1.3)	23.5 (1.3)	22.5 (1.2)	72124	23.6 (1.4)
Very small VLDL (%)	5359 (2659)	26.8 (2.2)	25.8 (2.1)	27.8 (1.8)	72151	27.6 (2.4)
IDL (%)	5359 (2659)	25.6 (0.9)	25.8 (0.9)	25.5 (0.9)	72148	26.7 (0.893)
Large LDL (%)	5359 (2659)	25.8 (1.9)	26.8 (1.9)	24.7 (1.2)	72137	25.9 (1.8)
Medium LDL (%)	5359 (2659)	28.7 (3.8)	30.7 (4.0)	26.8 (2.4)	71883	29.1 (3.7)
Small LDL (%)	5359 (2659)	31.7 (4.3)	34.0 (4.3)	29.4 (2.8)	71891	32.8 (4.3)
Very large HDL (%)	5359 (2659)	36.5 (9.5)	37.3 (10.0)	35.6 (8.9)	71701	45.4 (6.9)
Large HDL (%)	5359 (2659)	45.9 (5.6)	46.2 (5.0)	45.6 (6.1)	71097	47.9 (2.8)
Medium HDL (%)	5359 (2659)	45.9 (1.9)	46.4 (1.5)	45.5 (2.2)	71993	47.3 (2.1)
Small HDL (%)	5359 (2659)	54.2 (4.8)	56.6 (3.6)	51.7 (4.5)	72134	56.1 (3.8)
**Cholesteryl esters to total lipids ratio (CE%)**						
Extremely large VLDL (%)	5346 (2647)	15.4 (9.6)	15.3 (11.7)	15.5 (6.9)	67207	11.7 (4.5)
Very large VLDL (%)	5339 (2642)	16.3 (10.1)	16.1 (11.5)	16.5 (8.4)	63082	15.9 (7.2)

Large VLDL (%)	5349 (2650)	15.1 (5.9)	14.1 (6.1)	16.1 (5.5)	65528	17.6 (6.4)
Medium VLDL (%)	5358 (2658)	18.4 (4.6)	16.7 (4.4)	20.0 (4.3)	72028	20.0 (4.9)
Small VLDL (%)	5359 (2659)	26.7 (4.7)	25.2 (4.4)	28.2 (4.5)	72108	26.3 (5.0)
Very small VLDL (%)	5359 (2659)	35.1 (3.3)	35.2 (3.4)	35.0 (3.2)	72148	36.7 (3.7)
IDL (%)	5359 (2659)	44.0 (2.0)	43.3 (2.0)	44.7 (1.7)	72137	45.1 (2.5)
Large LDL (%)	5359 (2659)	44.7 (4.0)	42.4 (3.9)	46.9 (2.6)	72103	46.2 (3.5)
Medium LDL (%)	5359 (2659)	42.1 (7.5)	37.8 (7.4)	46.2 (4.8)	71836	43.0 (7.0)
Small LDL (%)	5359 (2659)	40.1 (7.4)	36.0 (7.1)	44.2 (5.1)	71846	40.6 (7.2)
Very large HDL (%)	5359 (2659)	45.3 (8.4)	44.9 (9.2)	45.6 (7.5)	71727	38.9 (5.7)
Large HDL (%)	5359 (2659)	37.2 (4.7)	37.7 (4.1)	36.7 (5.2)	71092	37.5 (2.6)
Medium HDL (%)	5359 (2659)	38.3 (2.5)	38.7 (2.3)	37.9 (2.7)	71984	38.4 (3.4)
Small HDL (%)	5359 (2659)	28.8 (4.9)	26.3 (3.9)	31.2 (4.7)	72118	28.3 (4.7)
**Free cholesterol to total lipids ratio (FC%)**						
Extremely large VLDL (%)	5346 (2647)	7.8 (2.6)	7.9 (3.4)	7.6 (1.6)	67231	6.6 (2.1)
Very large VLDL (%)	5339 (2642)	10.7 (5.2)	9.4 (5.3)	12.0 (4.8)	63096	11.9 (4.6)
Large VLDL (%)	5349 (2650)	9.9 (3.1)	9.0 (3.3)	10.9 (2.5)	65544	9.7 (3.6)
Medium VLDL (%)	5358 (2658)	12.0 (1.1)	11.7 (1.2)	12.3 (1.0)	72020	11.1 (1.4)
Small VLDL (%)	5359 (2659)	15.6 (0.9)	15.4 (0.8)	15.7 (0.9)	72124	14.1 (0.786)
Very small VLDL (%)	5359 (2659)	17.2 (1.2)	17.1 (1.3)	17.2 (1.2)	72143	15.7 (1.4)
IDL (%)	5359 (2659)	18.4 (1.5)	18.0 (1.5)	18.8 (1.4)	72128	17.6 (1.3)
Large LDL (%)	5359 (2659)	20.1 (1.4)	20.6 (1.5)	19.7 (1.1)	72132	19.5 (1.2)
Medium LDL (%)	5359 (2659)	21.7 (3.0)	23.4 (3.1)	20.1 (1.8)	71883	20.8 (2.5)
Small LDL (%)	5359 (2659)	21.1 (2.6)	22.5 (2.6)	19.7 (1.8)	71891	19.8 (2.2)
Very large HDL (%)	5359 (2659)	12.9 (1.9)	12.5 (2.0)	13.3 (1.7)	71717	12.0 (1.4)
Large HDL (%)	5359 (2659)	10.7 (2.0)	10.8 (1.8)	10.6 (2.2)	71097	9.7 (1.8)
Medium HDL (%)	5359 (2659)	10.3 (1.1)	10.0 (0.9)	10.5 (1.2)	71973	8.7 (0.864)
Small HDL (%)	5359 (2659)	12.0 (0.8)	12.3 (0.6)	11.8 (0.8)	72134	10.8 (0.767)
**Amino acids**						
Alanine (µmol/L)	5336 (2649)	286 (68)	287 (68)	284 (68)	72059	445 (77.1)
Glutamine (µmol/L)	5323 (2642)	414 (57)	415 (56)	413 (58)	71631	532 (77.5)
Glycine (µmol/L)	EDTA*	EDTA*	EDTA*	EDTA*	58606*	338 (62.6)
Isoleucine (µmol/L)	5353 (2656)	47 (15)	46 (14)	47 (15)	72059	63.0 (19.7)
Leucine (µmol/L)	5353 (2656)	59 (11)	60 (11)	59 (11)	72066	92.4 (22.9)
Valine (µmol/L)	5348 (2652)	142 (29)	143 (29)	141 (29)	71145	194 (44.1)

Phenylalanine (µmol/L)	5353 (2656)	46 (7)	46 (7)	46 (7)	72049	80.0 (12.6)
Tyrosine (µmol/L)	5352 (2655)	58 (11)	58 (11)	58 (11)	71969	60.2 (14.2)
Histidine (µmol/L)	5353 (2656)	44 (13)	44 (14)	44 (12)	71918	78.1 (13.1)
**Glycolysis and gluconeogenesis**						
Glucose (mmol/L)	Excluded*	Excluded*	Excluded*	Excluded*	31134**	4.4 (1.1)
Lactate (mmol/L)	Excluded*	Excluded*	Excluded*	Excluded*	31184**	1.5 (0.476)
Pyruvate (µmol/L)	EDTA*	EDTA*	EDTA*	EDTA*	18298**	84.5 (25.7)
Citrate (µmol/L)	5349 (2653)	97.752 (24)	97 (24)	98 (23)	31111**	113 (21.9)
Glycerol (µmol/L)	EDTA*	EDTA*	EDTA*	EDTA*	17130**	107 (56.7)
**Ketone bodies**						
Acetoacetate (µmol/L)	5353 (2656)	64 (48)	65 (49)	63 (47)	72140	43.2 (36.5)
Beta-hydroxybutyrate (µmol/L)	5336 (2647)	129 (94)	127 (90)	131 (98)	31051**	150 (114)
**Miscellaneous**						
Creatinine (µmol/L)	5352 (2655)	74 (18)	73 (18)	74 (18)	70426	59.2 (13.9)
Albumin (cu)	5356 (2658)	0.082 (0.006)	0.082 (0.006)	0.082 (0.006)	72077	0.094 (0.0098)
Acetate (µmol/L)	5352 (2656)	29 (31)	28 (33)	29 (28)	38375**	52.1 (41.7)
**Inflammation**						
Glycoprotein acetyls (mmol/L)	5353 (2656)	1.289 (0.2)	1.3 (0.2)	1.305 (0.2)	67654	1.4 (0.242)

Abbreviations: C: cholesterol; Cu: standardized concentration units; GlycA: Glycoprotein acetylation; HDL: high density lipoprotein; LDL: low density lipoprotein; MUFA: monounsaturated fatty acids; PUFA: polyunsaturated fatty acids; TG: triglycerides; VLDL: very low density lipoprotein.

Excluded* due to pre-analytic issue: Glucose and lactate were omitted from analyses due to severe glycolysis progression after sample collection, resulting in unphysiological concentrations.

EDTA*: Pyruvate, glycine, and glycerol were not quantifiable by NMR from EDTA plasma samples (PROSPER, INTERVAL, ALSPAC mums and kids, and CKB), due over to overlapping signals in the NMR spectra.

**: Numbers of individuals included in genetic analyses reduced, since metabolite concentration values were not available or low success rate of quantification, primarily in INTERVAL. The underlying reason was glycolysis progression after sample collection (glucose, lactate), signal overlap due to EDTA (glycerol, glycine, pyruvate), isopropyl alcohol (beta-hydroxybutyrate, acetate) or excess citrate in the plasma samples.

Mean concentrations of the metabolic measures and standard deviations (SD) were averaged across the eight cohorts used for genetic analyses.

All the 228 lipoprotein, lipid and metabolite measures were quantified using the Nightingale NMR metabolomics platform (Helsinki, Finland). The 14 lipoprotein subclass sizes were defined as follows: extremely large VLDL with particle diameters from 75 nm upwards and a possible contribution of chylomicrons, five VLDL subclasses (average particle diameters of 64.0 nm, 53.6 nm, 44.5 nm, 36.8 nm, and 31.3 nm), IDL (28.6 nm), three LDL subclasses (25.5 nm, 23.0 nm, and 18.7 nm), and four HDL subclasses (14.3 nm, 12.1 nm, 10.9 nm, and 8.7 nm). The mean size for VLDL, LDL and HDL particles was calculated by weighting the corresponding subclass diameters with their particle concentrations. Remnant cholesterol was defined as VLDL-cholesterol + IDL-cholesterol, which is equivalent to total-cholesterol - HDL-cholesterol - LDL-cholesterol.

Figure S1. Effects of statin treatment and genetic inhibition of PCSK9 on 228 metabolic traits.

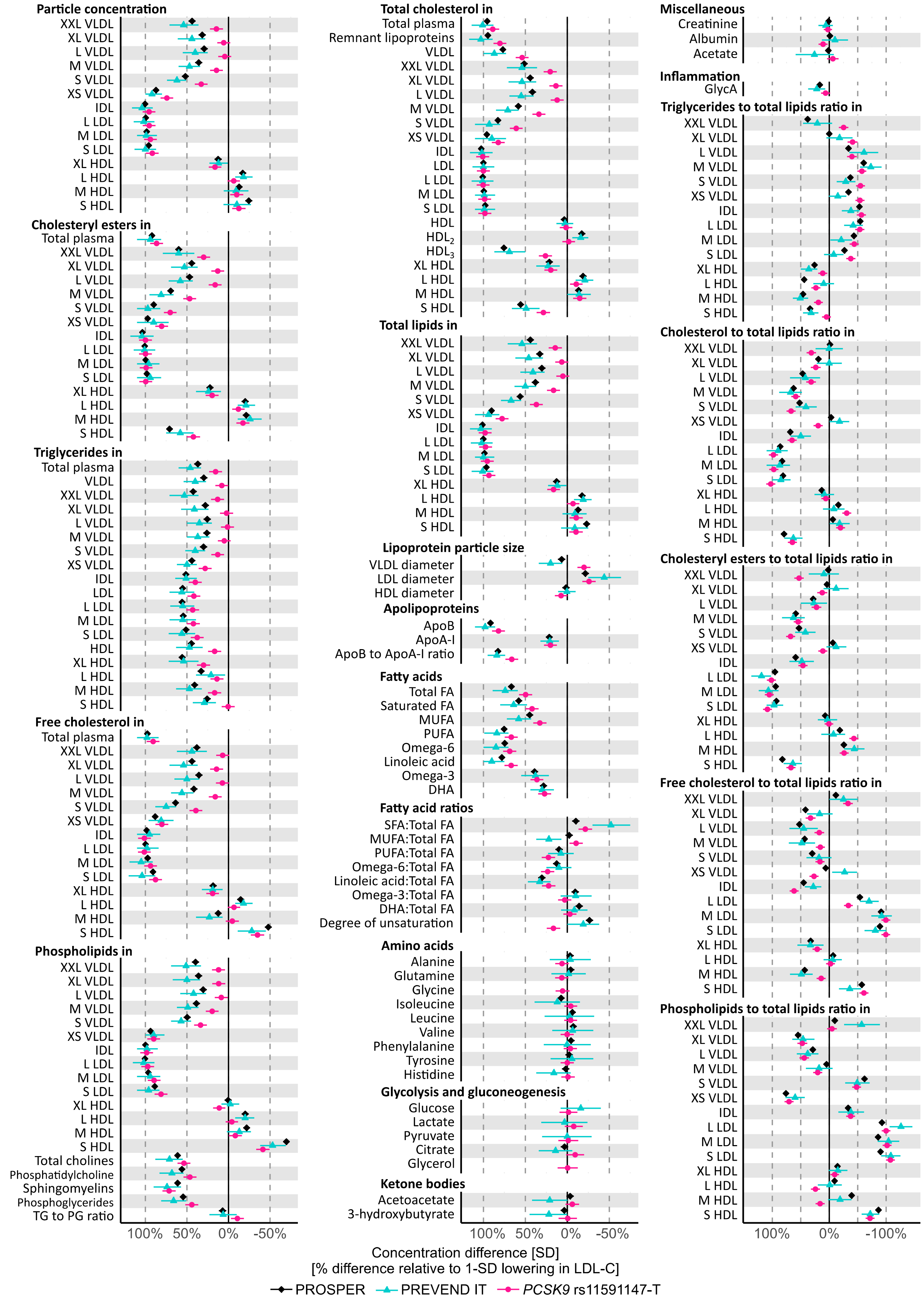

Results of the present study are compared with metabolic effects of pravastatin (40 mg/day) in PREVEND IT obtained using the same NMR metabolomics platform (Kofink et al, Circ Cardiovasc Genet. 2017;10:e001759). Effect estimates are shown in SD concentration units, scaled for each of the three analyses relative to the equivalent (1-SD) lowering effect on LDL-C. **Supplementary Tables S4 and S5** tabulates the results presented in this figure (Excel spreadsheet).

Figure S2. Consistency of metabolic effects of pravastatin 40 mg/day in PROSPER vs. PREVEND IT trials.

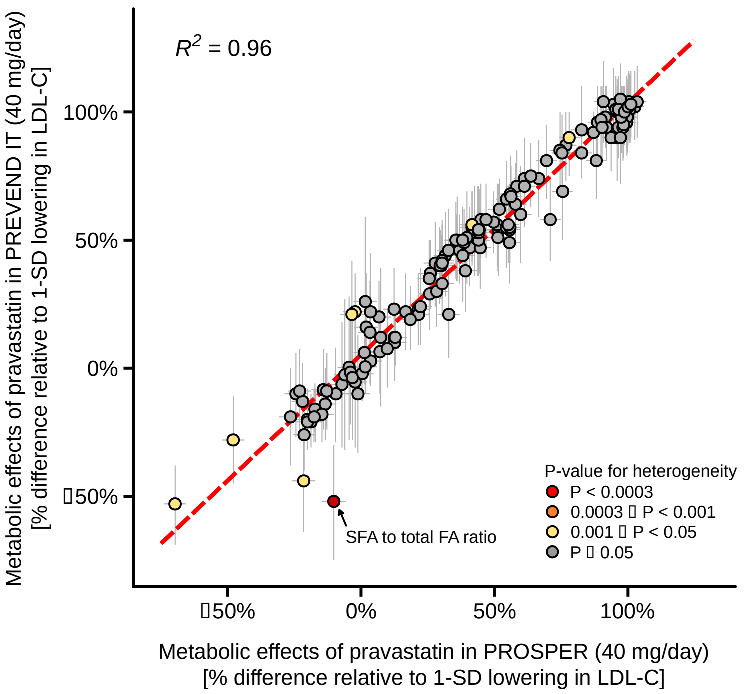

Effect sizes of each metabolic measure are given with 95% confidence intervals in grey vertical and horizontal error bars. Color coding for the metabolic measure indicates the P-value for heterogeneity between statin therapy in PROSPER and PREVEND IT. The consistency of the metabolic effects was summarized using R^2 goodness-of-fit for a subset of 153 metabolites (shown in the figure), excluding lipoprotein lipid ratios and five metabolites that were not available for the PROSPER trial (pyruvate, lactate, glucose, glycine, and glycerol; R^2=0.95 if estimated using all 223 measures available in both statin trials). The red dashed line denotes the linear fit between the metabolic effects (slope = 0.98).

Figure S3. Consistency of metabolic effects of *HMGCR* rs12916-T vs. (A) *PCSK9* rs11591147-T, and (B) statin therapy in the PROSPER trial.

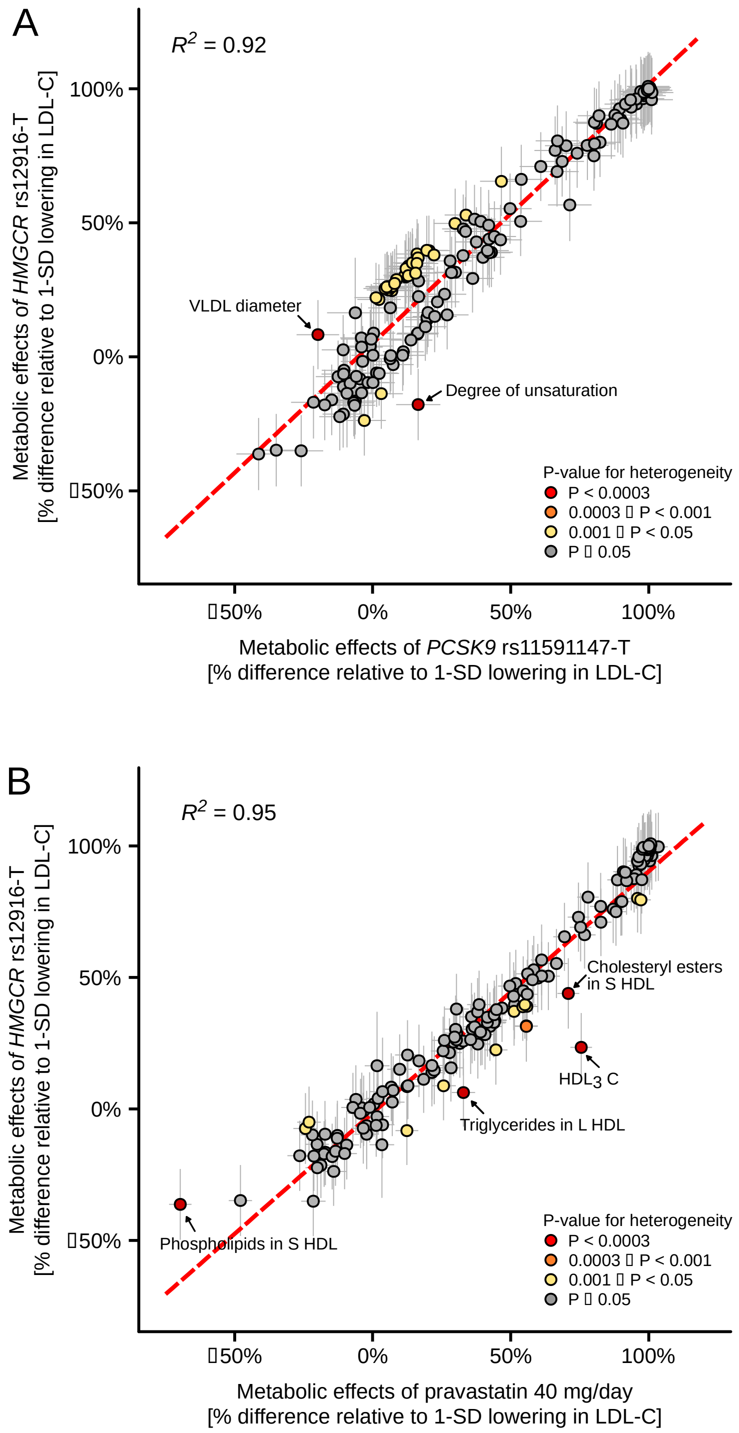

Effect sizes of each metabolic measure are given with 95% confidence intervals in grey vertical and horizontal error bars. Color coding for the metabolic measure indicates the P-value for heterogeneity between the metabolic effects of (A) *PCSK9* rs11591147-T vs. *HMGCR* rs12916-T (slope = 0.97), and (B) statin therapy in the PROSPER trial vs. *HMGCR* rs12916-T (slope = 0.92). The consistency of the metabolic effects was summarized using R^2 goodness-of-fit for a subset of 153 metabolites, excluding lipoprotein lipid ratios and five metabolites that were not available for the PROSPER trial (pyruvate, lactate, glucose, glycine, and glycerol). Similar R^2 were obtained if including all assayed metabolic measures in the estimation (R^2=0.93 for *PCSK9* vs *HMGCR* and R^2=0.93 for pravastatin in PROSPER vs. *HMGCR*). The red dashed lines denote the linear fits between the metabolic effects.

Figure S4. Metabolomic effects of statin treatment, *HMGCR* rs12916-T and *PCSK9* rs11591147-T.

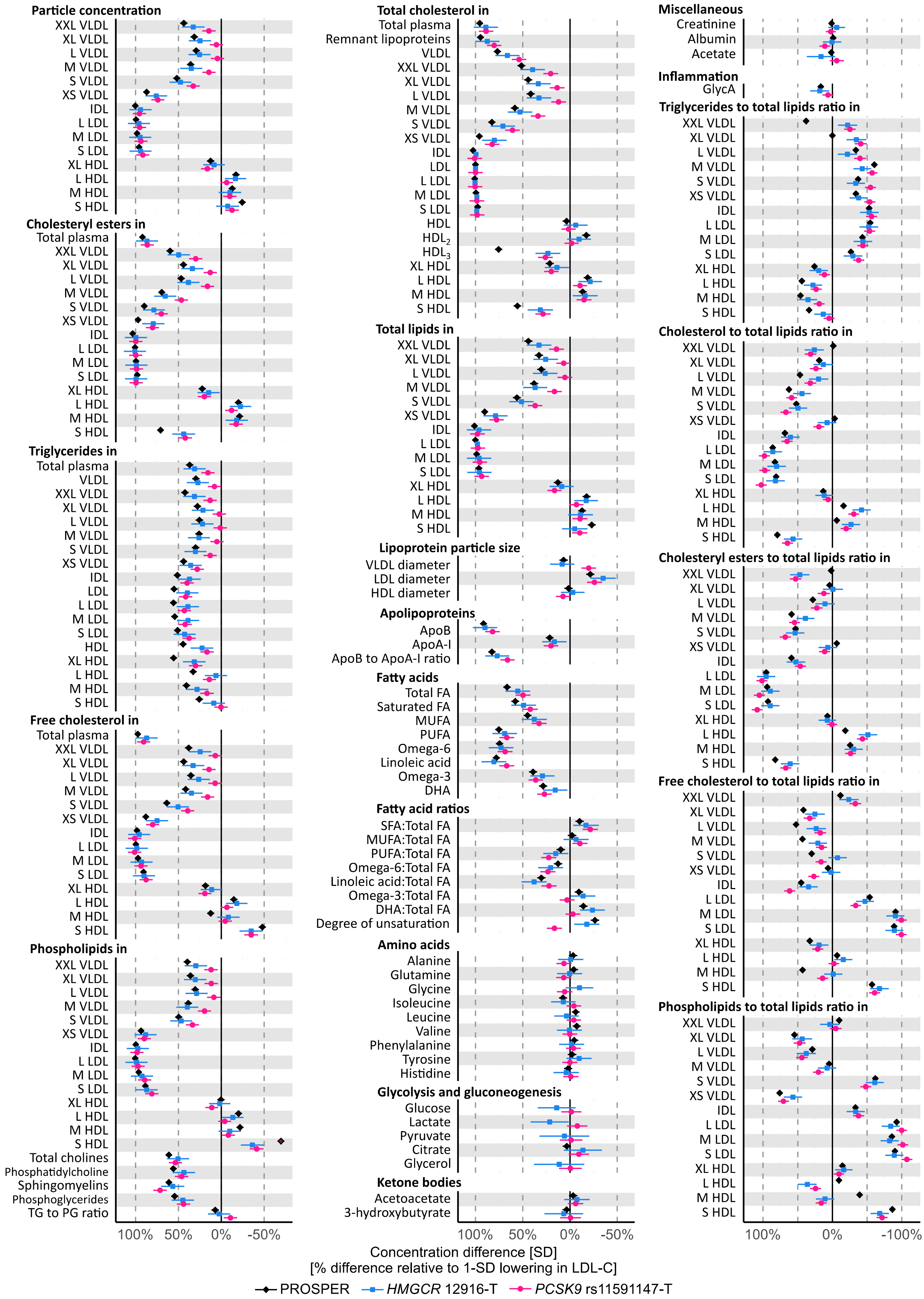

Comparison of the metabolic effects of genetic inhibition of HMGCR with those of genetic inhibition of PCSK9 and statin treatment in PROSPER. Effect estimates are shown in SD concentration units, scaled for each of the three analyses relative to the equivalent (1-SD) lowering effect on LDL-C. Error bars indicate 95% confidence intervals. Due to weaker LDL-C lowering effect of *HMGCR* rs12916-T, the power to detect statistical differences on individual measures was modest. **Supplementary Tables S4 and S5** tabulates the results presented in this figure (Excel spreadsheet).

Figure S5. Consistency of metabolomic effects of *PCSK9* rs11591147-T across the cohorts.

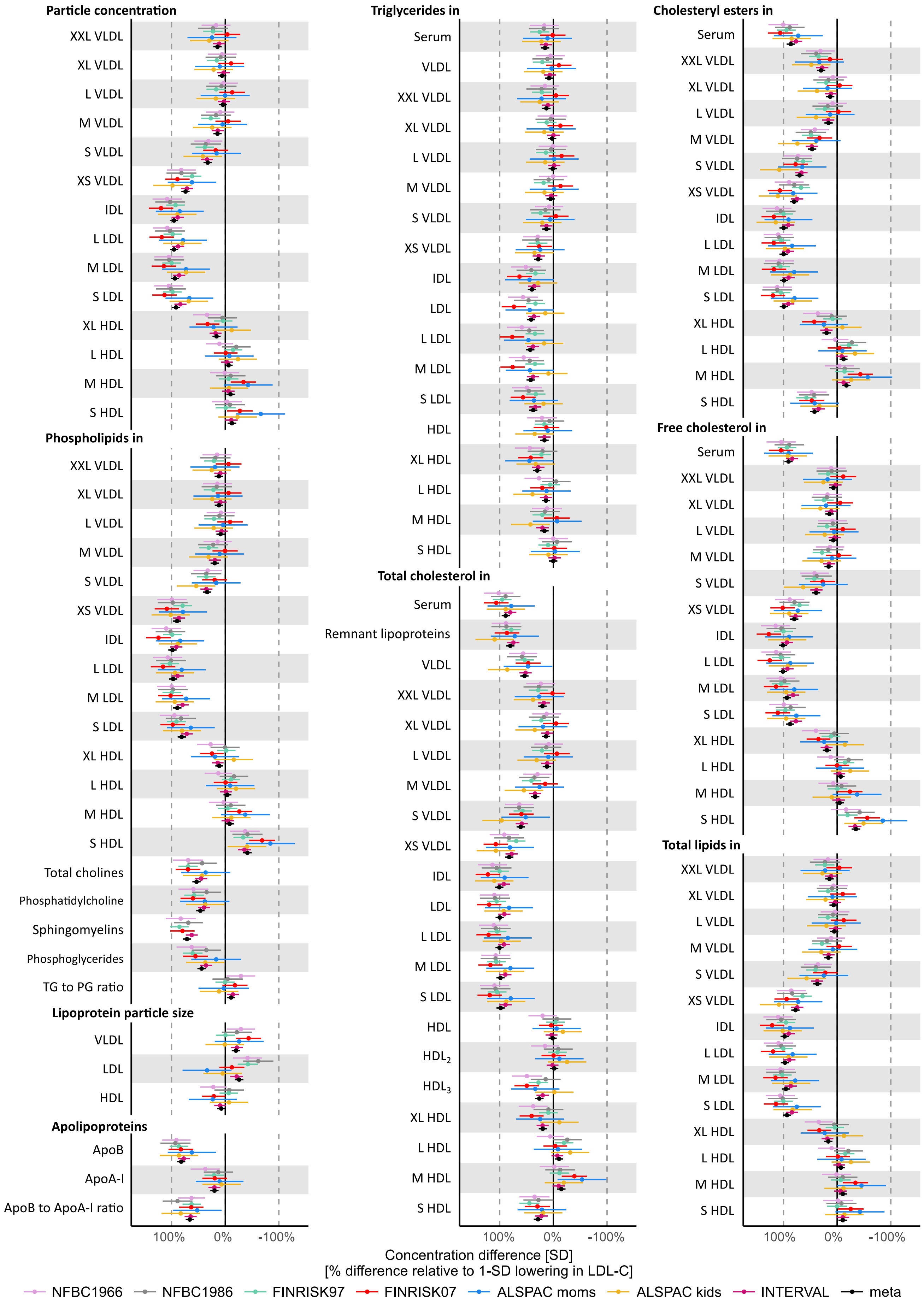

Metabolic effects of *PCSK9* rs11591147-T across the study populations used for genetic analyses. The genetic effects were determined in each of the cohorts separately and scaled with respect to the meta-analyzed effect on LDL-C. Error bars indicate 95% confidence intervals. Due to the low allele frequency in the Chinese population, *PCSK9* rs11591147-T was not present in the China Kadoorie Biobank dataset.

Figure S5. Consistency of metabolomic effects of *PCSK9* rs11591147-T across the cohorts (Continued).

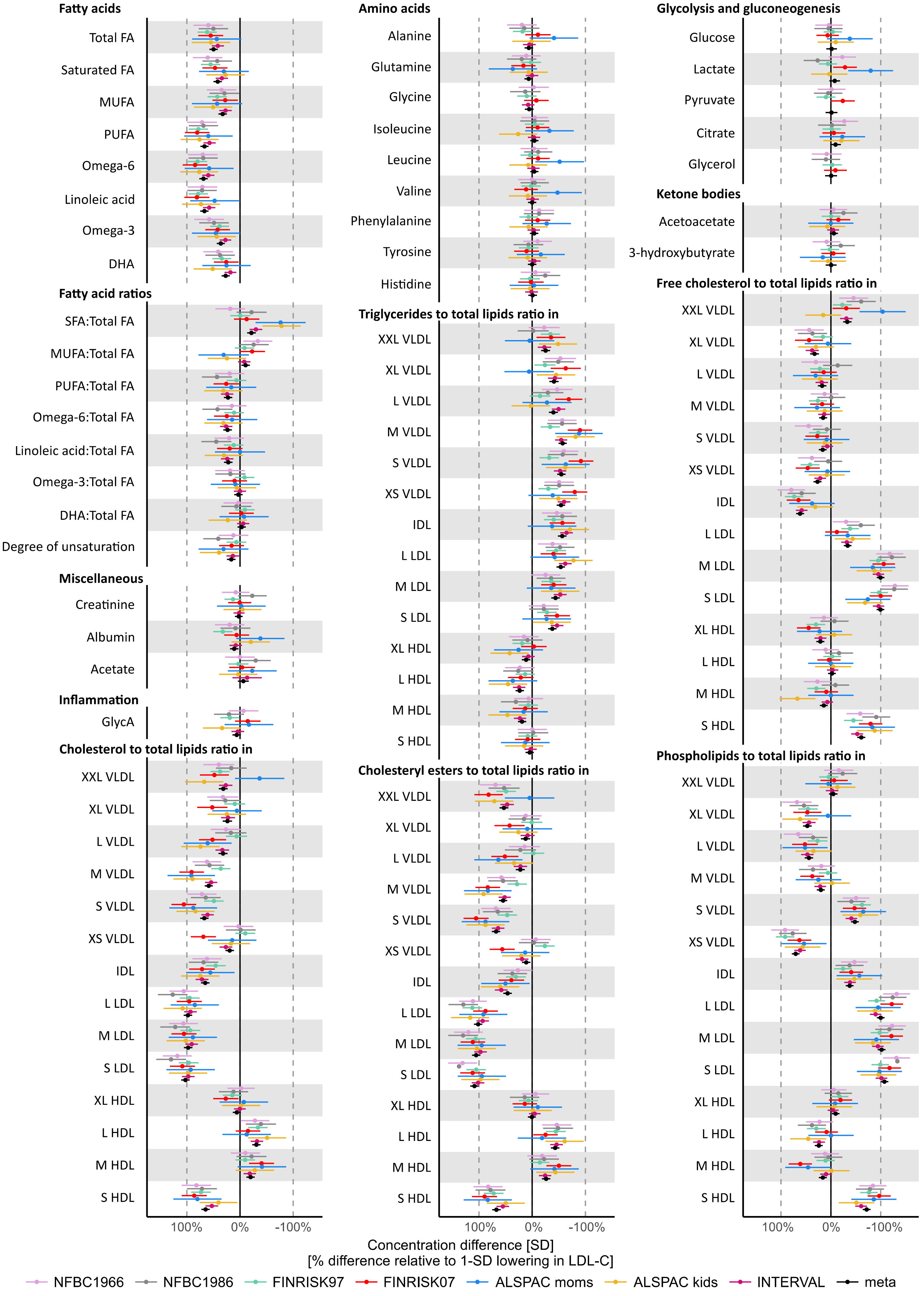

Figure S6. Consistency of metabolic effects of the main instrument *PCSK9* rs11591147-T with other *PCSK9* variants.

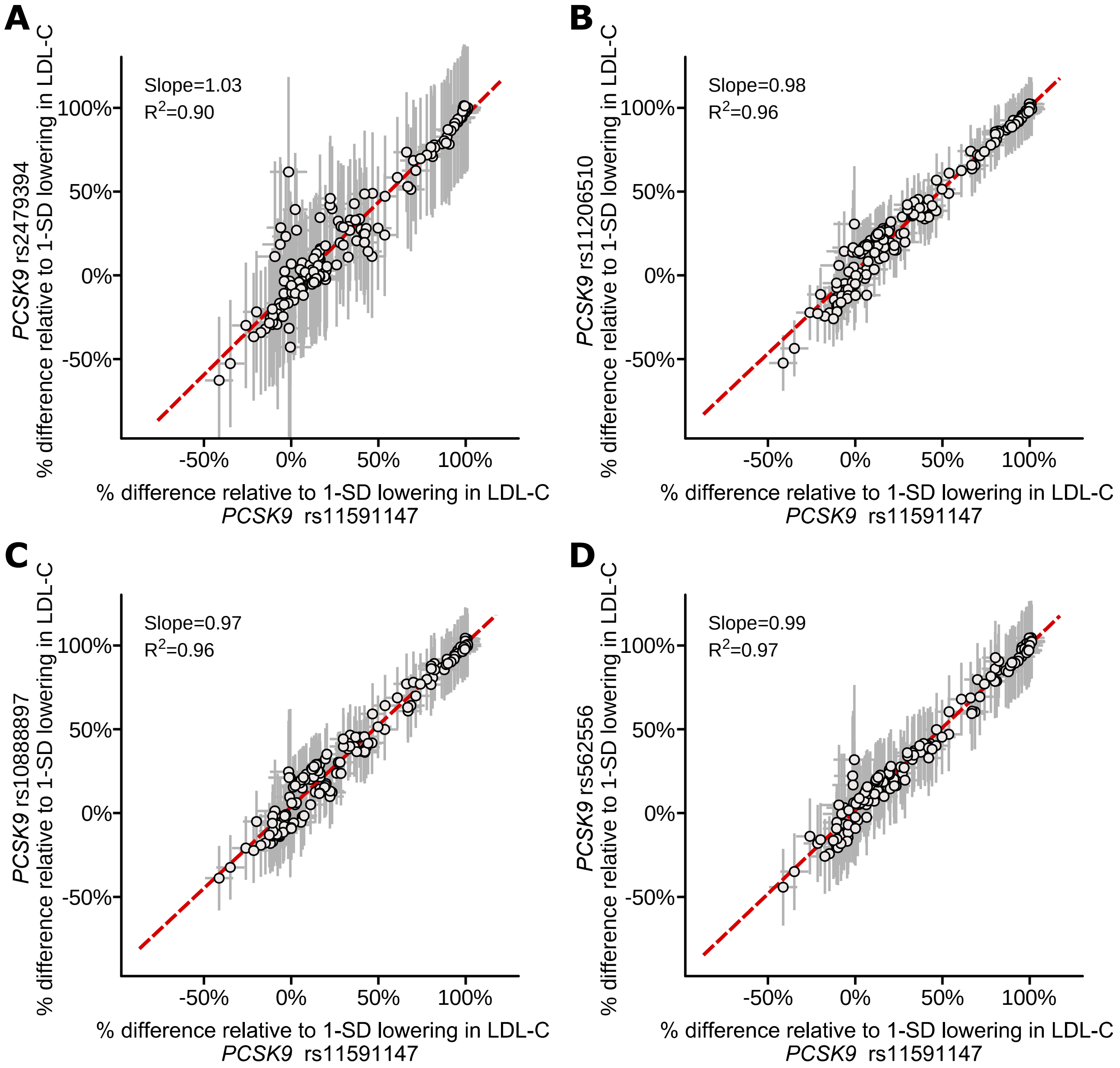

Assessment of metabolic effects of **A**) rs2479394, **B**) rs11206510, **C**) rs10888897 and **D**) rs562556 in the *PCSK9* locus in comparison to main instrument *PCSK9* rs11591147. These variants have previously been used in Mendelian randomization studies on *PCSK9* and all display low linkage disequilibrium ($R^2 \leq 0.2$) with the main instrument rs11591147. Effect sizes of each metabolic measure is given with 95% confidence intervals in grey vertical and horizontal error bars. The correspondence of metabolic effects was summarized using R^2 goodness-of-fit for a subset of 158 metabolic measures (excluding lipoprotein lipid ratios). The red dashed line denotes the linear fit between the metabolic effects.

Figure S7. Percentage differences in metabolite concentrations in pravastatin versus placebo group in PROSPER.

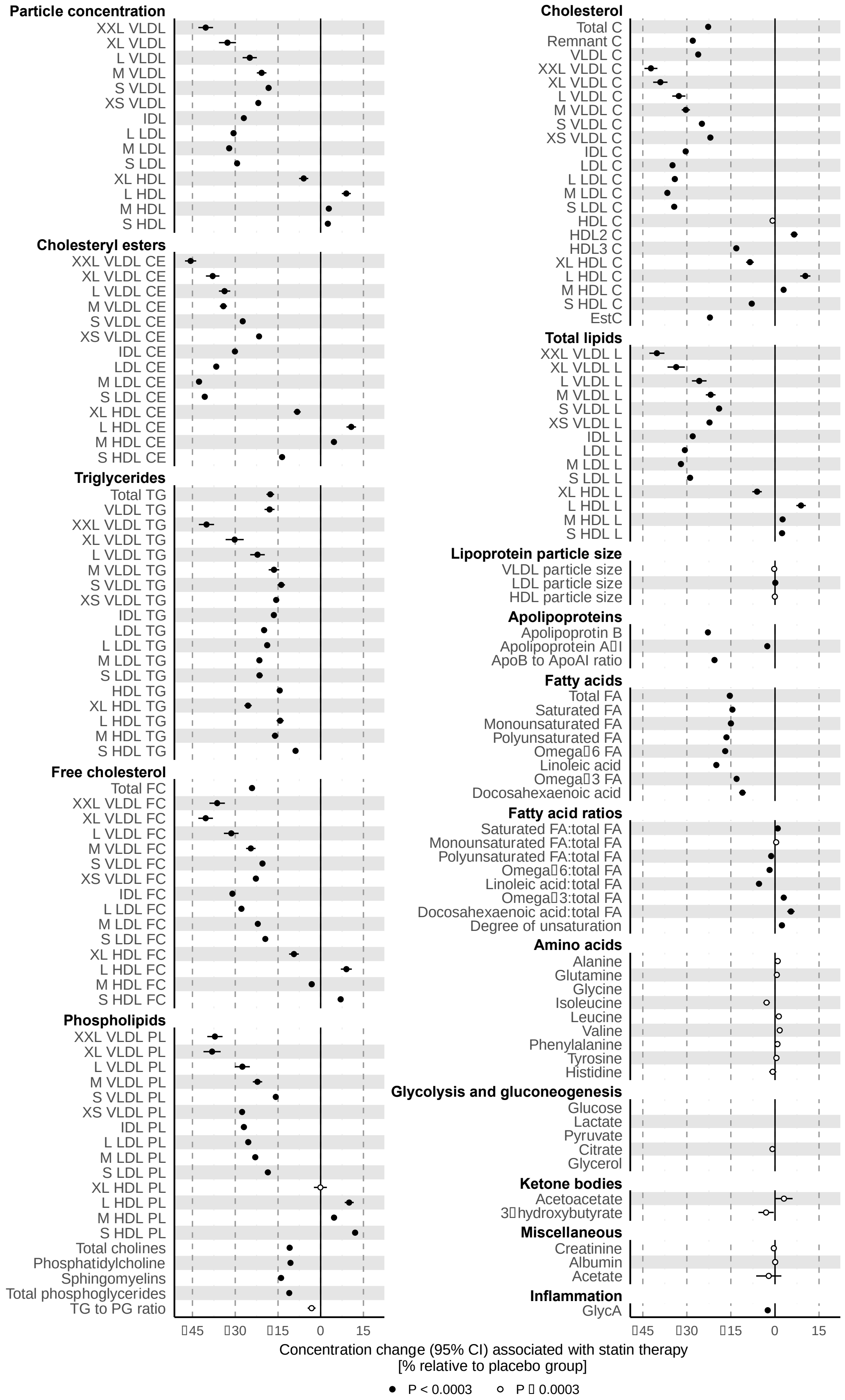

The percentage difference was calculated from the mean values of pravastatin group relative to placebo group in the PROSPER trial at 6 months after randomization. Data from a single time point do not allow assessment of change among individuals from baseline, however the randomization should ensure that there are limited between-group differences at baseline. The pattern of lipid changes deviates primarily from the SD-scaled results presented in **Figures 3-6** for metabolic measures with very low concentration, such as the largest VLDL subclasses and their lipid content. The numerical results are tabulated in **Supplementary Table S3** (separate Excel file).
